# Prodrug-like Quinone Containing Two Redox-Active Centers Induces Ferroptosis in Prostate Cancer Cells with Cytotoxic Action Partially Depending on NAD(P)H Quinone Dehydrogenase 1 (NQO1)

**DOI:** 10.1021/acsomega.5c11250

**Published:** 2026-08-25

**Authors:** Eleicy N. Mendoza, Maria Fernanda Madrid, Pedro M. S. da Costa, Serena Castelli, Fabio Ciccarone, Maria R. Ciriolo, Bruno C. Cavalcanti, Emmanuel S. Marinho, Jacilene Silva, Eveline M. Bezerra, Roner F. da Costa, Renata G. Almeida, Joyce C. de Oliveira, Francisco Washington A. Barros-Nepomuceno, Manoel O. de Moraes Filho, Eufrânio N. da Silva Júnior, Claudia Pessoa, Carlos R. K. Paier

**Affiliations:** † Department of Physiology and Pharmacology, Drug Research and Development Center, Universidade Federal do Ceará, Fortaleza, Ceará 60430-275, Brazil; ‡ Department for the Promotion of Human Science and Quality of Life, Universita Telematica San Raffaele Roma, Via di Val Cannuta 247, 00166 Rome, Italy; § Department of Biology, 9318 University of Rome Tor Vergata, Via della Ricerca Scientifica, 00133 Rome, Italy; ∥ Laboratory of Bioprospecting and Monitoring of Natural Resources, Center of Science and Technology, 67843 Universidade Estadual do Ceará, Fortaleza, Ceará 60714-903, Brazil; ⊥ Postgraduate Program in Materials Science and Engineering, 74384 Universidade Federal Rural do Semi-Arido, Mossoró, Rio Grande do Norte 59625-900, Brazil; # Department of Chemistry, Institute of Exact Sciences, 113014 Universidade Federal de Minas Gerais, Belo Horizonte, Minas Gerais 31270-901, Brazil; ∇ Institute of Health Sciences, Universidade da Integração Internacional da Lusofonia Afro-Brasileira, Redenção, Ceará 62790-000, Brazil

## Abstract

Naphthoquinones are redox-active compounds with antitumor potential, often linked to NQO1 overexpression in cancer cells. This study evaluated the cytotoxic activity of two synthetic 1,4-naphthoquinones, compound **1** and its dual redox analog, compound **4**, against NQO1-overexpressing DU-145 prostate cancer cells through integrated *in vitro*, enzymatic, and computational (docking and quantum) analyses. Compound **4** exhibited over 2-fold greater cytotoxicity against DU-145 cells compared to nontumor MRC-5 cells, while maintaining low hemolytic activity. Evidence of membrane damage, ROS generation, lipid peroxidation, and reversal by deferoxamine indicated ferroptosis induction by **4**, whereas **1** triggered caspase-3-dependent apoptosis. Both caused ROS-mediated DNA damage attenuated by NAC. Docking and enzymatic assays confirmed partial dependence on NQO1, with **4** showing greater ROS production despite lower enzymatic activity. Overall, compound **4** highlights the potential of dual-redox quinone platforms to induce ferroptosis, providing a viable lead candidate for novel antineoplastic drug discovery in prostate cancer and other malignancies.

## Graphical abstract

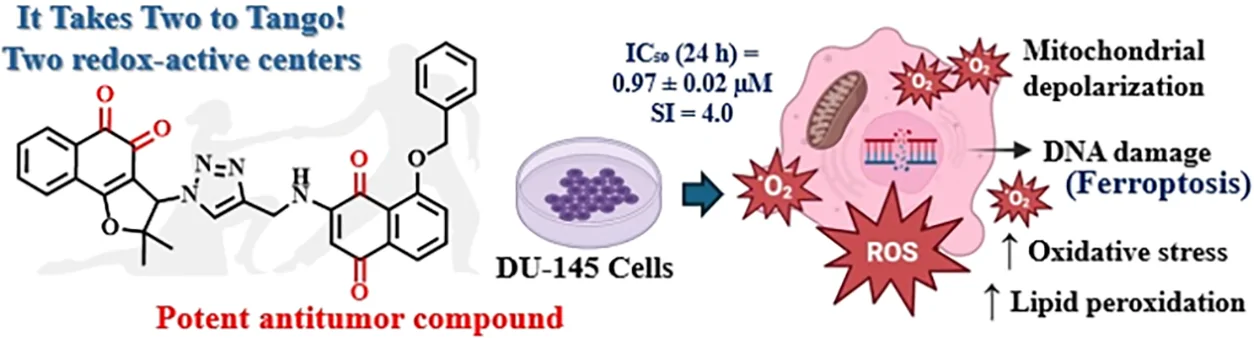

## Introduction

Prostate cancer (PCa) typically advances gradually, resulting in abnormal enlargement of the prostate gland, which can substantially affect quality of life due to the presence of slowly developing tumors. PCa has the potential to induce metastatic processes in distant locations in the body, commonly including bones, the pelvic region, the lumbar vertebrae, the bladder, the rectum, and even the brain.^1^ In 2020, PCa ranked as the second most diagnosed malignancy and the fifth leading cause of cancer-related deaths among men. According to the American Cancer Society’s projections for 2024, approximately 299,010 new cases of prostate cancer are expected in the United States, resulting in an estimated 35,250 deaths.^2^ The high epidemiological burden positions prostate cancer as a crucial focus of research, driving the development of novel therapeutic strategies, particularly in light of emerging chemoresistance to standard treatment regimens.^3^

In recent years, quinones have been extensively studied due to their diverse pharmacological properties. Referred to as “privileged structures” in medicinal chemistry, these compounds can interact with multiple targets with high affinity, facilitating the discovery of novel molecules by appropriately tailoring their structure with functional groups.^4^ For several decades, quinones have served as a source of cytotoxic compounds found in various medications such as daunorubicin, doxorubicin, and mitoxantrone, which are clinically used in cancer therapy.^5^

Depending on their chemical structure, quinones can be categorized into benzoquinones, naphthoquinones, and anthraquinones based on the number of fused rings or the position of carbonyl groups, resulting in specific pharmacological actions.^6^ Many naphthoquinones (e.g., lapachol, shikonin, and naphthazarin) have shown potential efficacy for further development as anticancer drugs,^7^ as their cytotoxicity has been associated with various mechanisms such as the inhibition of human DNA topoisomerases I and II and the production of reactive oxygen species (ROS) that lead to oxidative stress.^5^ Furthermore, quinones are rapidly converted to hydroquinone/semiquinone species in the presence of NQO1 or cytochrome P-450, which then revert to quinones to form a redox cycle. Quinones with high reversibility undergo repeated oxidation–reduction cycles without significant degradation, thereby inducing the production of high amounts of ROS. The excessive accumulation of ROS leads to severe oxidative stress, causing cellular damage at the DNA and protein levels, damaging cell membranes, and ultimately leading to cancer cell death. Consequently, there has been an increasing emphasis on developing novel quinone-based therapeutic agents capable of exploiting tumor-associated metabolic vulnerabilities, such as elevated NQO1 expression across a broad spectrum of malignancies.^4^

Ferroptosis is a recently discovered iron-dependent process characterized by the activation of diverse signaling pathways in response to oxidative stress, culminating in regulated cell death marked by lipid peroxidation.^8^ It is closely associated with many diseases, especially tumor growth, since tumor cells exhibit a higher dependency on iron compared to normal cells, fueling their rapid proliferation. The emergence of ferroptosis has provided a novel perspective on tumor progression, as increasing evidence indicates that ferroptosis leads to tumor growth inhibition.^9^ Previous studies have shown that the induction of ferroptosis or the regulation of ferroptosis-related genes may represent a new anticancer strategy for prostate cancer,^8,9^ now recognized as the Achilles’ heel of cancer cells.^10^ These discoveries offer promising avenues for anti-PCa therapies centered around ferroptotic cell death induction.^9,11^

Previous studies conducted by our group have reported the synthesis of 47 novel quinone-based derivatives utilizing click chemistry, followed by their cytotoxic evaluation, which revealed significant potential against cancer cell lines.^12^ Furthermore, it has been established that the incorporation of two redox centers into a quinone molecule enhances its cytotoxic potential, leading to increased antiproliferative efficacy.^13^ Based on these results, compound **4**, was selected for further investigation due to its particularly high antitumor potential observed in our previous study.^12^ Building on these findings, the present study aimed to investigate the *in vitro* antitumor cytotoxicity and mechanism of action of compound **4** (Scheme 1, a) synthetic naphthoquinone synthesized via Cu­(I)-catalyzed azide–alkyne cycloaddition (CuAAC), and evaluated against prostate cancer cells (DU-145). To elucidate its biological activity, a comprehensive study was conducted comprising *in silico* analyses, molecular docking simulations, and enzymatic assays involving NQO1. This integrative approach aimed to provide a detailed understanding of its potential mechanisms of action and structure–activity relationships.

**Scheme 1:**
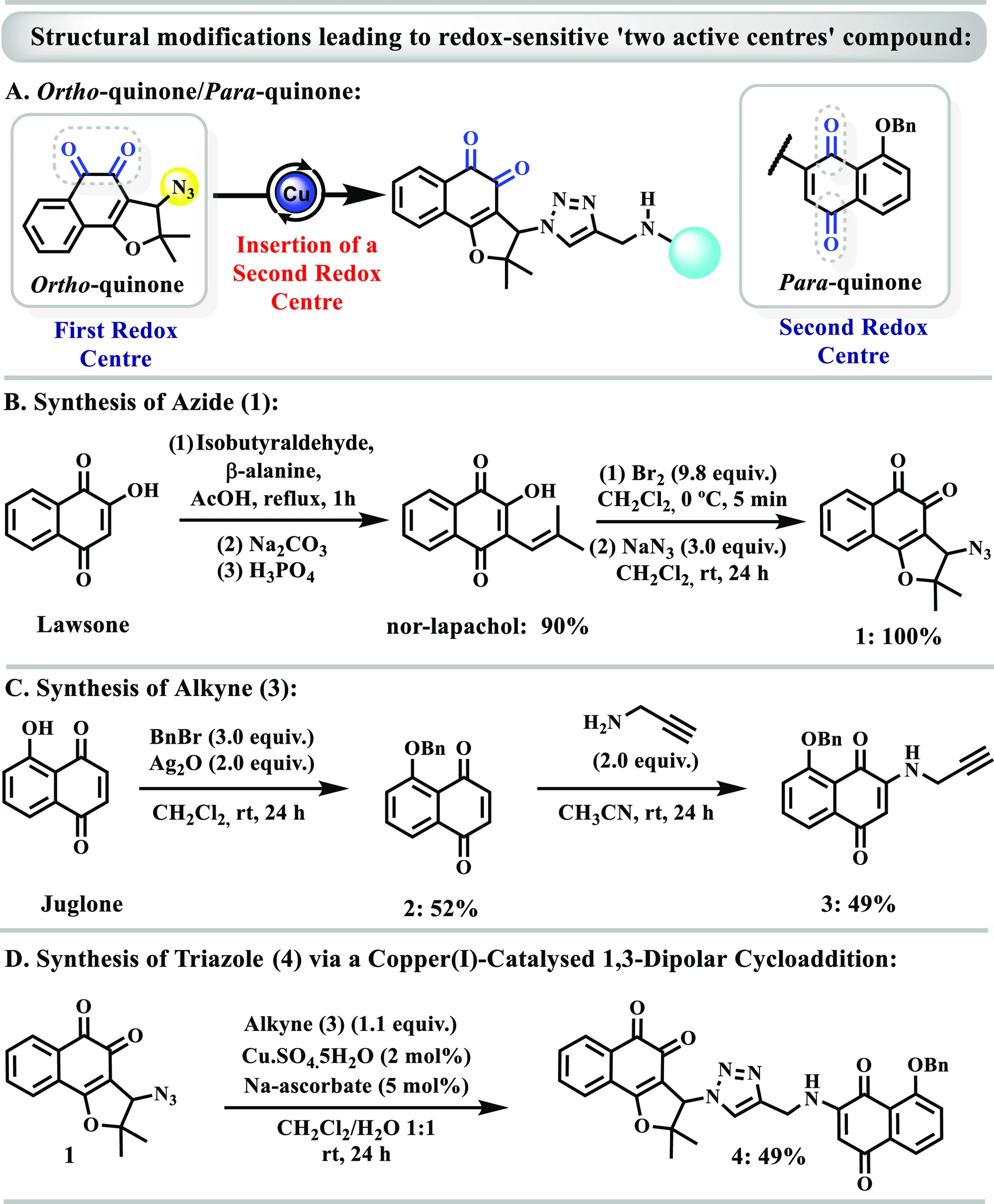
Synthesis of 4 via CuAAC

## Results and Discussion

The Results and Discussion section is structured in two parts for clarity and coherence. First, the experimental results are presented, emphasizing the key synthetic, characterization, biological, and computational findings. This is followed by a detailed discussion that offers a thorough interpretation of the data and their correlation with theoretical insights and relevant literature.

### Chemistry

***Synthesis of Azide (1)*** **:** The synthesis of the compound **4** was based on the azide-functionalized quinone moiety. For this purpose, the azide derived from the *ortho*-quinone was obtained, as shown in Scheme 1B. Compound **1** was prepared from nor-lapachol through a bromide-mediated cycloaddition followed by nucleophilic substitution with sodium azide.^15^ This sequence afforded the desired ortho-quinone in quantitative yield. ***Synthesis of Amino-Alkyne (3)*** **:** To enable the desired reaction with the azide shown above, an aminoalkyne was designed from the corresponding A-ring–modified naphthoquinone. Compound 5-Benzyloxy-1,4-naphthoquinone **2** was synthesized from juglone through a Lewis acid–catalyzed nucleophilic substitution. Subsequently, the A-ring–modified quinone was subjected to amination with propargylamine (Scheme 1C), following a previously reported procedure.^16^ This transformation afforded A-ring–modified alkyne quinones, which are promising candidates for exhibiting relevant bioactive properties once the target products are obtained. ***Synthesis of Triazole (4) via CuAAC*** **:** To construct the compound **4**, a click chemistry reaction was employed to join two quinone moieties. In this process, the azide **1** and the corresponding alkyne **3** underwent a copper­(I)-catalyzed 1,3-dipolar cycloaddition, affording a highly stable 1,2,3-triazole-based product. The azide **1** was prepared from nor-lapachol via a cycloaddition reaction with bromide, followed by nucleophilic substitution with sodium azide, providing the desired product in good yield. The alkyne precursor of the corresponding triazole was synthesized from a functionalized 1,4-naphthoquinone through amination with propargylamine, furnishing the alkyne.^14^ Following a previously reported methodology,^17^ the reaction between **1** (azide) and the alkyne was carried out in the presence of copper­(II) sulfate pentahydrate (2 mol %) as the catalyst and sodium L-ascorbate (5 mol %) as the reducing agent, using a dichloromethane/water mixture (1:1) as the solvent system, to afford compound **4**. **1**, the parent molecule, was used in all experiments as a reference in order to assess whether the introduction of an additional redox center could effectively enhance the cytotoxic potential of the compound (Scheme 1D).

### Cytotoxicity and Ferroptosis Induction

Initially, MTT bioassays were conducted to determine the cytotoxicity level of the compounds against tumor cells. Figure 1 shows the IC_50_ values following different incubation times (24, 48, and 72 h) and the greater cytotoxic effect of **4** (IC_50_ < 2 μM) against DU-145 cells when compared with **1** and the positive control β-lapachone. The observed cytotoxic effect did not present a time-dependent profile. Additionally, compound **4** exhibited reduced toxicity toward nonmalignant MRC-5 cells relative to DU-145 cells, yielding a favorable selectivity index (SI > 3.5) for this cell pair across all evaluated time points. Viability data were normalized to negative control (DMSO 0.5%) and β-lapachone was used as positive control.

**Figure 1:**
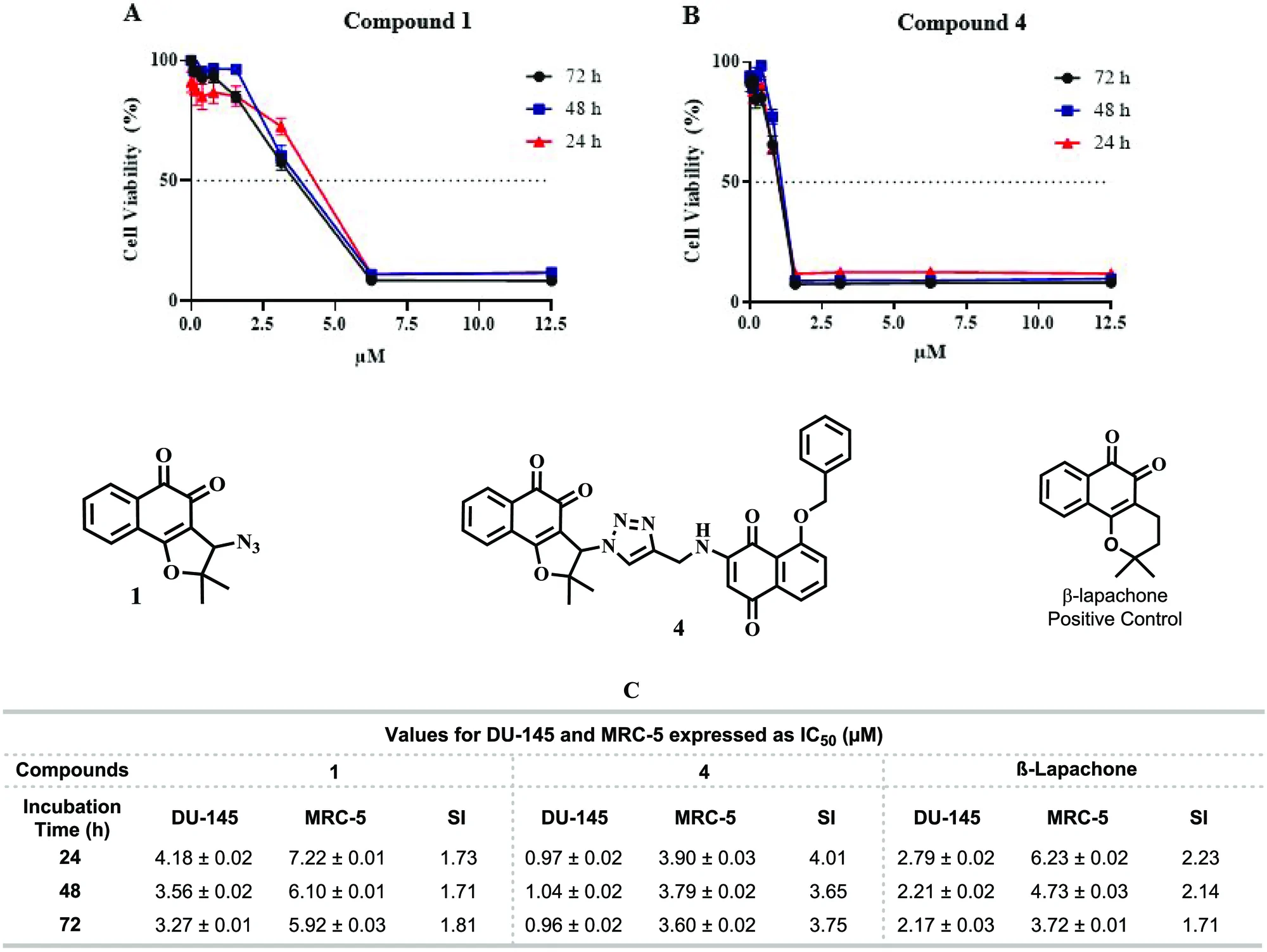
Cytotoxic effect of **1** and **4** at different concentrations (0.098 to 12.5 μM) against DU-145 and MRC-5 cells after 24, 48, and 72 h of incubation using MTT Assay. Graphical representation of DU-145 cell viability versus (A) **1** and (B) **4** concentrations. (C) half maximal inhibitory concentration (IC_50_ ± S.E.M.) from three independent experiments performed in triplicate calculated by prism 8.0 (GraphPad/Intuitive software for science, San Diego, CA). Viability data were normalized to negative control (DMSO 0.5%). β-Lapachone (0.098 to 12.5 μM) was used as positive control of the test. The selectivity index (SI) is the ratio of the IC_50_ values of the treatments on MRC-5 cells to those in DU-145 cells.

Morphological changes in DU-145 cells were assessed using light microscopy (Figure 2). Following a 24-h incubation period, **1** induced the formation of apoptotic bodies, while **4** caused an increase in cytoplasmic size. The positive control showed signs of membrane integrity loss and chromatin condensation. In contrast, the negative control (DMSO 0.5%) revealed DU-145 cells with a mesenchymal morphology characterized by visible cytoplasm and a well-defined nucleus (Figure 2A). Flow cytometric analysis demonstrated a notable decrease in cellular density in a concentration-dependent manner (Figure 2B), and the obtained values were 14.58 ± 0.25 × 10^4^ and 9.05 ± 0.17 × 10^4^ cells/mL for **1** (2 and 4 μM), and for **4** (0.5 and 1 μM), 13.71 ± 0.09 × 10^4^ and 8.23 ± 0.15 × 10^4^ cells/mL, respectively. A significant decrease in cellular viability, assessed through membrane integrity flow cytometric analysis, was observed across all tested concentrations, particularly with compound **1** at 4 μM (62.04 ± 0,70%), compound **4** at 0,5 μM (83.37 ± 2,70%) and **4** at 1 μM (48.83 ± 2.99%), compared to negative control (94.27 ± 1.02%). β-Lapachone (3 μM) also induced a significant reduction in cellular density (10.96 ± 0.24 × 10^4^ cells/mL) and viability (81.99 ± 2.02%).

**Figure 2:**
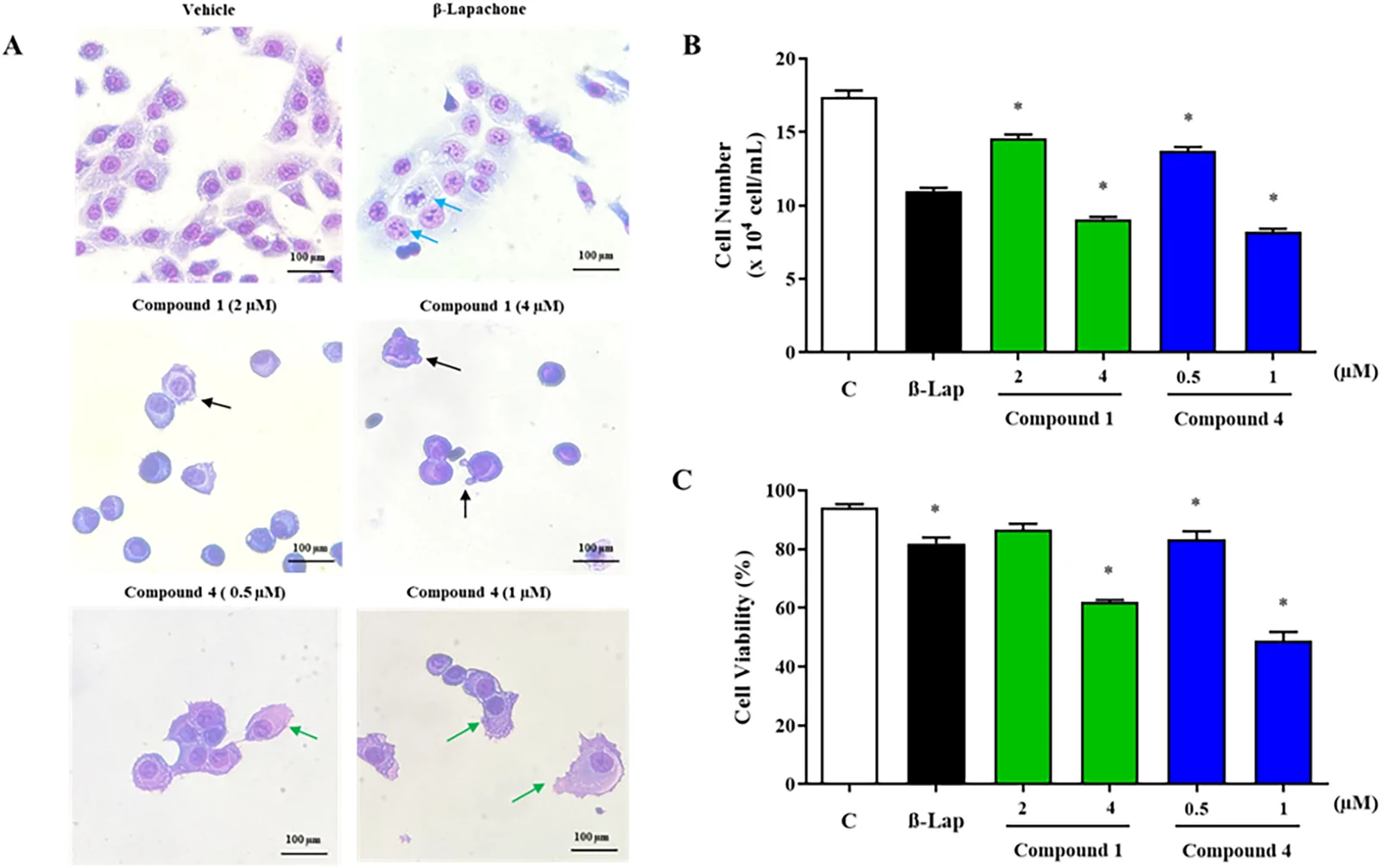
Morphological changes (A), trypan blue counting (B), and viability by flow cytometry (C) of DU-145 human tumoral cells following treatment for 24 h with different concentrations of **1** (2 and 4 μM), **4** (0.5 and 1 μM), the positive control (β-Lapachone 3 μM), and the negative control (DMSO 0.5%). In the figure, black arrows indicate apoptotic bodies, blue arrows represent chromatin condensation and green arrows indicate increased cytoplasmic size. The cells were analyzed using light microscopy at 200× magnification. Data are presented as mean ± SEM from three independent experiments performed in triplicate and were calculated using Prism 8.0 (GraphPad/Intuitive Software for Science, San Diego, CA). Statistical analyses for multiple comparisons were conducted using one-way ANOVA followed by Tukey’s test. The significance level of *p < 0.05 was considered statistically significant.

In sequence, the hemolytic activity was analyzed using the mouse erythrocyte lysis assay. Both naphthoquinones tested, **1** and **4**, were not able to cause hemolysis (EC_50_ > 200 μM) suggesting that their cytotoxic effect involves a specific mechanism of action other than direct damage to the cell membrane.

To investigate potential cell death pathways induced by these naphthoquinones, inhibitors of specific cell death types were employed as pre- and cotreatments, and incubated with compounds **1** and **4** at their respective IC_50_ values for 24 h (Figure 3). Interestingly, it was noted that each inhibitor used led to a slight increase in cell viability compared to the control without inhibitor. However, a notable and statistically significant increase (**p < 0.001 and ***p < 0.0001) in cell viability was observed in cells treated with **4** in conjunction with the iron chelator deferoxamine (DFO), used at concentrations of 50 μM (48.73 ± 6.70% cell viability) and 100 μM (62.54 ± 6.05% cell viability). In these instances, cell viability increased more than 3-fold compared to cells treated solely with **4** (14.18 ± 3.41%). Conversely, cells treated with **1** showed a significant increase (*p < 0.05) in cell viability when simultaneously treated with the caspase **3** inhibitor (41.59 ± 7.88%). Additionally, there was an increase in viability of cells treated with compound **1** in the presence of DFO at 100 μM (36.90 ± 1.0%), which was not statistically significant when compared to cells treated with compound **1** alone (17.10 ± 0.49%). These findings suggest the occurrence of apoptosis in cells treated with **1**, while indicating another type of cell death in cells treated with **4**, specifically an iron-dependent cell death pathway. In cells treated with doxorubicin (positive control) in combination with Z-VAD-FMK (92.14 ± 3.71%) and 3-methyladenine (3-MA) (96.89 ± 3.11%), a significant increase in cell viability was observed compared to cells treated only with doxorubicin (70.37 ± 3.15%). This increase in cell viability with doxorubicin and the mentioned inhibitors suggests that this anticancer drug induces cell death through autophagy, necroptosis, and apoptosis, as reported in the literature.

**Figure 3:**
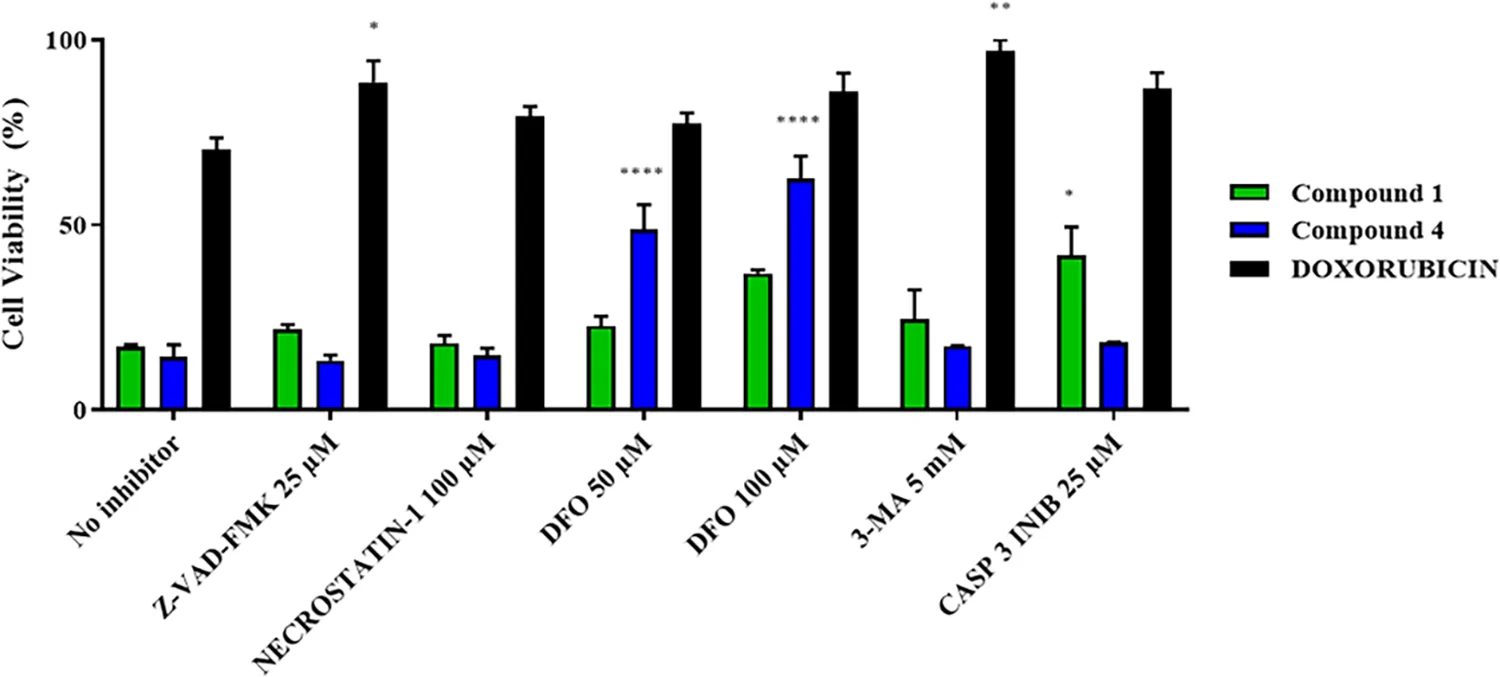
Cell viability of DU-145 cells treated with **1** (4 μM) and **4** (4 μM) for 24 h in the presence of cell death pathway inhibitors using MTT Assay. Z-VAD-FMK: caspase inhibitor; Necrostatin-1: RIPK1 inhibitor; DFO: iron chelator; 3-MA: PI3K inhibitor; CASP 3 INIB: apoptosis inhibitor. Data are presented as mean ± SEM of at least three independent experiments performed in triplicate. Statistical significance was determined by comparing corresponding groups of treatments and negative control (untreated with cell death inhibitor). Doxorubicin (3 μM) was used as positive control of the test. Significance levels were denoted as * *p* < 0.05, ** *p* < 0.01, *** *p* < 0.001, **** *p* < 0.0001. Mean comparison was performed by ANOVA followed by Tukey’s post-test (GraphPad/Intuitive Software for Science, San Diego, CA).

### Induction of Oxidative Stress

To verify the involvement of reactive oxygen species (ROS) generation in the mechanism of action of the naphthoquinones, DU-145 cells were pretreated with the antioxidant N-acetyl-l-cysteine (NAC; 5 mM), and the cytotoxic effects of the test compounds were determined by the MTT assay after a 24 h of incubation. As we can see in Table 1, **4** exhibited a significant reduction in its cytotoxicity (an approximately 3-fold increase in its initial IC_50_ value, from 0.971 ± 0.011 μM to 3.279 ± 0.299 μM). Similarly, β-lapachone also exhibited a decrease in its cytotoxic effect in NAC-pretreated cells (an increase in IC_50_ from 2.790 ± 0.163 μM to 7.565 ± 0.350 μM). Notably, **1** did not have its cytotoxicity altered.

**Table 1:** Cytotoxic Effect of **1** and **4** against DU-145 Cells with and without Pre-Treatment with NAC, after 24 h of Exposure Using MTT Assay

	IC_50_ (μM)	
Compound	DU-145	DU-145 + NAC pretreatment (5 mM)
**1**	4.18 ± 0.07	4.83 ± 0.02
**4**	0.97 ± 0.01	3.28 ± 0.30
β-Lapachone	2.79 ± 0.16	7.56 ± 0.35

In sequence, fluorescence microscopy using CM-H2DCFDA was employed to directly assess reactive oxygen species (ROS) levels after 3, 6, and 24 h of exposure to the naphthoquinones investigated (Figure 4). the positive control, hydrogen peroxide (H_2_O_2_), exhibited a statistically significant increase in fluorescent cells per field compared to the negative control, at 6 h (12.95 ± 0.90) and 24 h (19.09 ± 4.67%). Compound **4** induced ROS, resulting in a statistically significant increase in fluorescent cells at all three exposure times: 3 h (22.06 ± 0.89%), 6 h (39.70 ± 3.62%), and 24 h (18.01 ± 4.25%). Conversely, **1** did not have its cytotoxicity altered.

**Figure 4:**
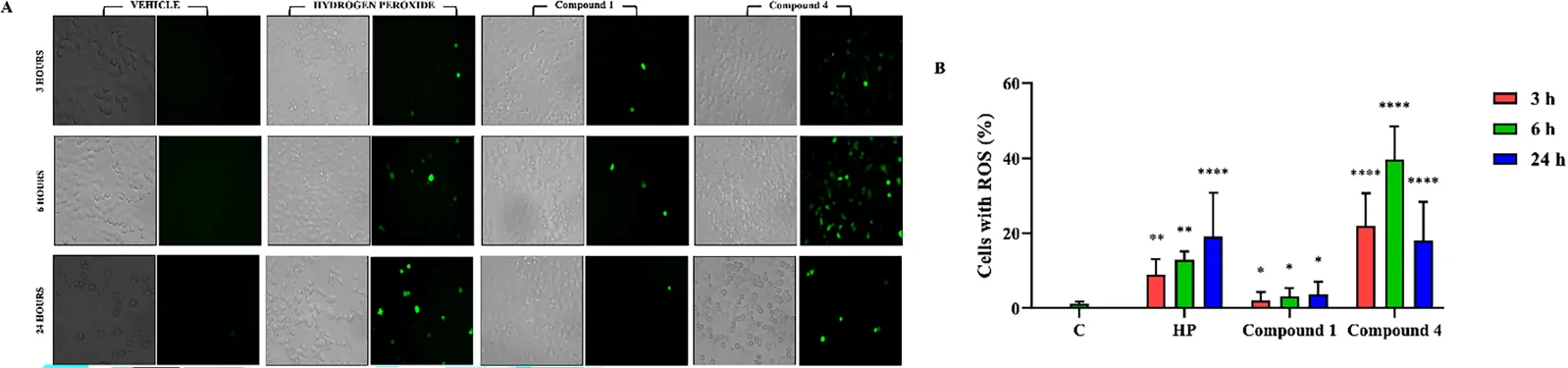
Determination of ROS production by fluorescence microscopy in DU-145 cells after 3, 6, and 24 h of treatment with **1** (4 μM), **4** (1 μM), hydrogen peroxide (HP, positive control 200 μM), and negative control (DMSO 0.5%). (A) Representative micrographs of each treatment. Cells were analyzed using bright-field and fluorescence microscopy with 200× magnification. (B) Data represent the mean ± SEM of three independent experiments conducted in triplicate. Mean comparison was performed using ANOVA followed by Tukey’s post-test (GraphPad Prism 8.0). Statistical significance was determined by comparing corresponding groups of treatments with the negative control, denoted as * *p* < 0.05, ** *p* < 0.01, *** *p* < 0.001, and **** *p* < 0.0001.

As the peak of ROS generation occurred after 6 h of exposure, this time of incubation was included when evaluating mitochondrial depolarization (Figure 5). After a 6-h incubation, mitochondrial membrane depolarization was observed with compound **1** at 4 μM (20.61 ± 1.70%), and compound **4** at 0.5 μM (22.64 ± 1.08%) and 1 μM (46.51 ± 1.93%). After 24 h of exposure, compound **1** at 4 μM (22.94 ± 2.96%), and compound **4** at 0.5 μM (16.21 ± 1.80%) and 1 μM (26.06 ± 1.55%), also induced mitochondrial depolarization. The reduced mitochondrial depolarization at 24 h compared to 6 h likely reflects progression of the death process. While depolarization is an early ROS-associated event, by 24 h many affected cells may have advanced to later stages of damage, decreasing the detectable depolarized population. The reduction in mitochondrial potential in the first hours of treatment is common, as mitochondria are involved in the first stages of the cell death process.^14^

**Figure 5:**
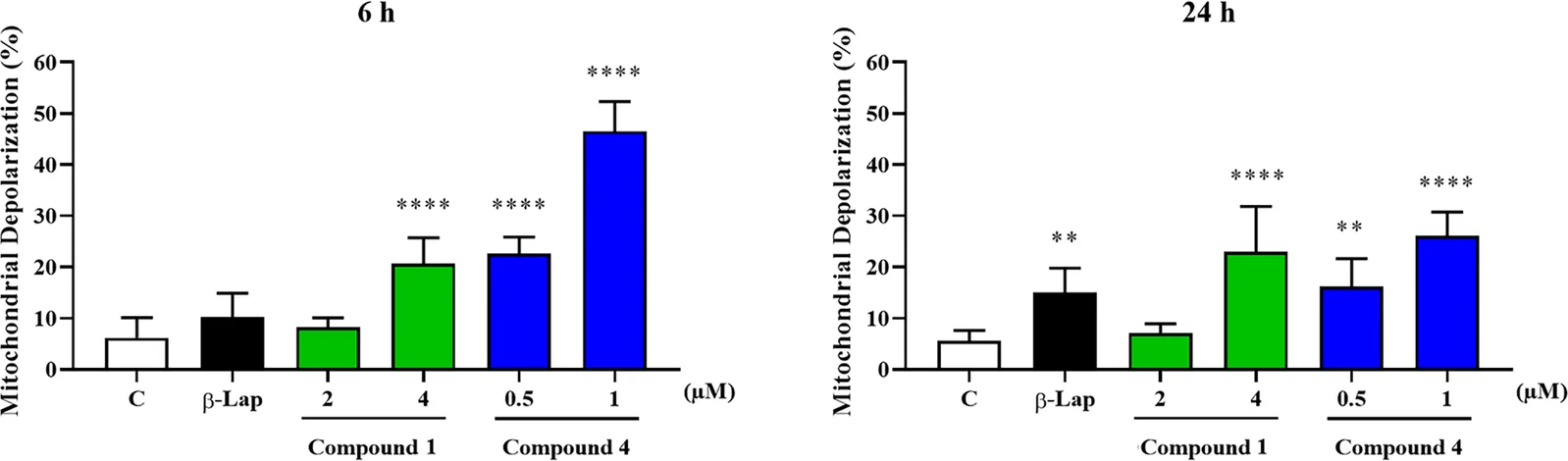
Mitochondrial depolarization of DU-145 cells after 6 and 24 h of incubation with **1** (2 and 4 μM) and **4** (0.5 and 1 μM) by flow cytometry. Data are presented as mean ± SEM from three independent experiments performed in triplicate and calculated using Prism 8.0 (GraphPad/Intuitive Software for Science, San Diego, CA). Statistical analyses for multiple comparisons were conducted using one-way ANOVA followed by Tukey’s test. Statistical significance was determined by comparing corresponding groups of treatments with the negative control, denoted as ** *p* < 0.01 and **** *p* < 0.0001. β-lapachone (3 μM) was used as a positive control and DMSO 5% (vehicle) as a negative control.

Fluorescence microscopy using C11-Bodipy sensor was used to evaluate lipid peroxidation, with red staining denoting nonoxidized lipids and green indicating oxidized lipids (Figure 6). Treatment with **4** (1 μM for 6 h) induced intense lipid peroxidation, as evidenced by the heightened intensity of green staining in treated cells. Additionally, small green spots corresponding to oxidized lipid droplets were observed within the cytoplasm. The oxidation rate (calculated as the ratio of oxidized fluorescence to nonoxidized fluorescence) for cells treated with **4** was determined to be 1.51 ± 0.28, a significant increase compared to the mean rates observed in the negative control (0.13 ± 0.01) and cells treated with 1 (0.15 ± 0.03).

**Figure 6:**
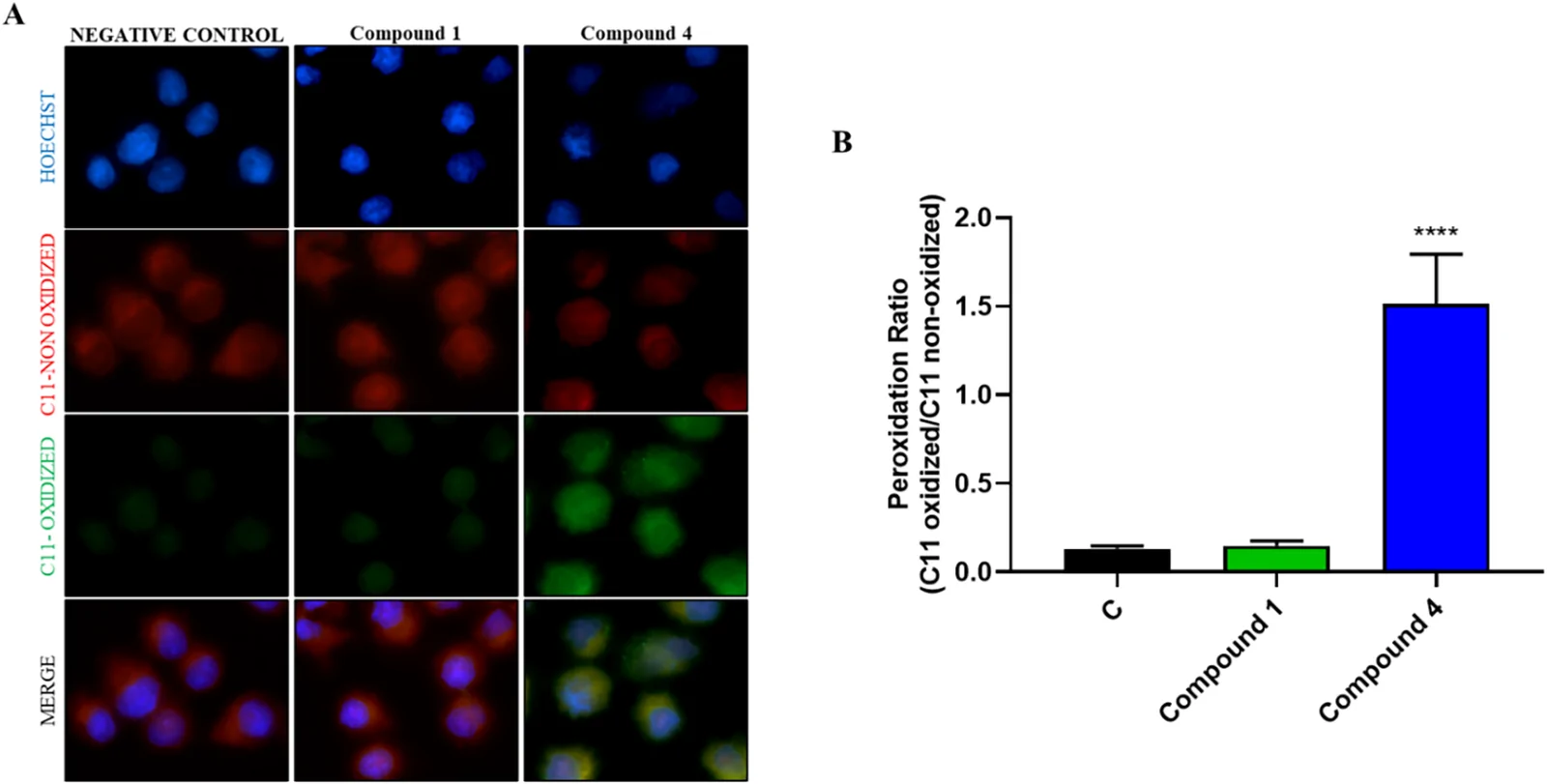
Lipid peroxidation induction in DU-145 cells treated with **1** (4 μM) and **4** (1 μM) after 6 h of treatment by fluorescence microscopy using C11-BODIPY. (A) Representative images of DU-145 cells treated (B) Quantification of the C11-BODIPY oxidation rate. Mean comparison was performed using ANOVA followed by Tukey’s post-test (GraphPad Prism 8.0). Statistical significance was determined by comparing between treatment group and negative control (C, DMSO 0.5%), denoted as **** *p* < 0.0001. C11-BODIPY fluorescence red (591 nm) in the absence of lipid peroxidation and green (510 nm) in the presence of lipid peroxidation. The oxidation rate is calculated as the quotient between the fluorescence intensity at 510 nm and the fluorescence intensity at 591 nm.

Data are presented as half maximal inhibitory concentration (IC50 ± S.E.M.) from three independent experiments performed in triplicate calculated by Prism 8.0 (GraphPad/Intuitive Software for Science, San Diego, CA). Each IC50 was obtained from a nonlinear regression of the cytotoxicity curves. Viability data were normalized to control group (DMSO 0.4%).

### DNA Damage

Initially, DNA damage was assessed using flow cytometry (Figure 7A). After a 24-h incubation, statistically significant differences were observed in cells treated with compound **1** at 4 μM (20.49 ± 1.26%), and **4** at 0.5 μM (10.20 ± 0.79%) and **1** μM (20.70 ± 1.12%), compared to the negative control (7.68 ± 1.20%). Additionally, pretreatment with 5 mM NAC, led to a reduction in DNA fragmentation across all groups. These findings suggest that NAC has the potential to attenuate DNA fragmentation induced by the study molecules.

**Figure 7:**
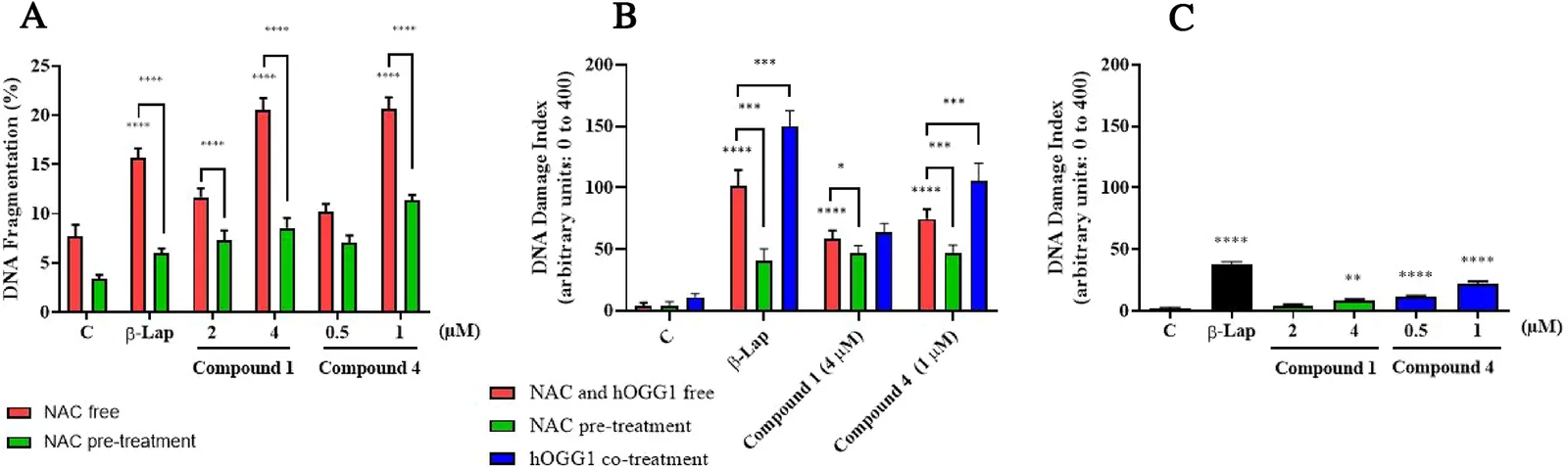
DNA damage in DU-145 cells after 24 h of treatment with **1** and **4**. (A) DNA fragmentation by flow cytometry, (B) comet assay in alkaline conditions and (C) neutral conditions. The data represent the mean ± SEM of at least three independent experiments performed in triplicate. For DNA fragmentation, ten thousand events were recorded for each experiment. Pretreatment with NAC (5 μM) was performed for 4 h. For alkaline comet assay, coincubation with hOGG1 enzyme (1:500) was also conducted. Mean comparison was performed using ANOVA followed by Tukey’s post-test (GraphPad Prism 8.0). Statistical significance was determined by comparing between treatment group and negative control (C, DMSO 0.5%) or correspondent group, denoted as * *p* < 0.05, ** *p* < 0.01, *** *p* < 0.001, and **** *p* < 0.0001. β-lapachone (3 μM) was used as a positive control and DMSO 5% (vehicle) as a negative control.

DNA damage was also assessed employing comet assay. Under alkaline conditions (Figure 7B), compound **1** induced significant DNA damage at 4 μM (58.44 ± 2.27 damage index) and compound **4** at 1 μM (74.66 ± 2.59 damage index), when compared to the negative control (4.22 ± 0.79 damage index). NAC pretreatment significantly diminished the DNA damage caused by compound **1** at 4 μM (46.88 ± 2.01 damage index) and by compound **4** at 1 μM (47.11 ± 2.11 damage index). Coincubation with hOGG1 enzyme (1:500) had a significantly enhanced impact on DNA, presenting a damage index of 63.55 ± 2.44 and 105.22 ± 4.88 after treatment with compound **1** at 4 μM and compound **4** at 1 μM, respectively. Under neutral conditions (Figure 7C), compound **1** also promoted DNA damage at 4 μM (8.44 ± 0.93 damage index), and compound **4** at 0.5 μM (11.11 ± 1.07 damage index) and 1 μM (22.44 ± 1.47 damage index). Similarly, β-lapachone induced DNA damage under both alkaline (101.11 ± 4.44 damage index) and neutral (37.88 ± 2.03 damage index) experimental conditions, which was diminished by NAC pretreatment (40.88 ± 3.20 damage index) but intensified by the hOGG1 enzyme (1:500) (150.00 ± 4.17 damage index).

### NQO1 Activity-Dependent Cytotoxic Action (MTT Assay)

To assess the involvement of NQO1 in the mechanism of action, the compounds were evaluated in DU-145 cells, a representative model of a high-burden cancer displaying high NQO1 expression, and HL-60 cells, which exhibit negligible to undetectable NQO1 levels. The cell assays were performed in the presence and absence of the selective NQO1 inhibitor dicoumarol (50 μM). The cell lines were chosen based on the expression profile described in The Human Protein Atlas database. As observed in Table 2, coincubation with dicumarol promoted a reduction in the cytotoxic effect induced by naphthoquinones in DU-145 cells, illustrated by an increase in the IC_50_ value from 4.182 ± 0.071 μM to 8.290 ± 0.250 μM for **1** and 0.971 ± 0.011 μM to 2.025 ± 0.335 μM for **4**. Similar profile was also observed for the positive control (β-lapachone). Conversely, the cytotoxic activity of the naphthoquinones against HL-60, a lineage that does not express NQO1, remained approximately unchanged after treatment with the dicoumarol inhibitor.

**Table 2:** Cytotoxic Effect of **1** and **4** (0.04 to 12.5 μM) in DU-145 and HL-60 Cells, in the Presence and Absence of Dicoumarol 50 μM (DIC), after 24 h of Treatment Using MTT Assay

	IC_50_ (μM)			
Compound	DU-145		HL-60	
	DIC (−)	DIC (+)	DIC (−)	DIC (+)
**1**	4.18 ± 0.07	8.29 ± 0.25	3.41 ± 0.05	3.57 ± 0.16
**4**	0.97 ± 0.01	2.02 ± 0.33	1.25 ± 0.14	1.41 ± 0.02
β-Lapachone	2.79 ± 0.16	7.22 ± 0.27	3.29 ± 0.14	4.06 ± 0.16

Data are presented as half maximal inhibitory concentration (IC50 ± S.E.M.) from three independent experiments performed in triplicate calculated by Prism 8.0 (GraphPad/Intuitive Software for Science, San Diego, CA). Each IC50 was obtained from a nonlinear regression of the cytotoxicity curves. Viability data were normalized to control group (DMSO 0.4%).

### NQO1 Assays

The enzymatic assay revealed an activity of 99.97 ± 1.10% for **1**, while molecule **4** promoted a lower enzymatic activity of 76.96 ± 4.46%. This difference was statistically significant compared to β-lapachone, the positive control, which serves as a substrate molecule of NQO1 (Figure 8A). To determine the thermal stability of the complex (protein–ligand) the thermal shift assay was performed, using dicoumarol, a competitive inhibitor, was used as control and found to form a more stable complex with NQO1 than the studied molecules, with a melting temperature of 57.67 ± 0.08 °C, followed by β-lapachone, with 52.22 ± 0.15 °C, both results were statistically significant (****p < 0.0001) compared to the NQO1 protein alone. Subsequently, compound **4** formed a complex with a melting temperature of 51.78 ± 0.09 °C, and finally, the complex of **1** exhibited a melting point of 51.44 ± 0.05 °C, which did not significantly differ from the melting point of NQO1 alone (Figure 8B).

**Figure 8:**
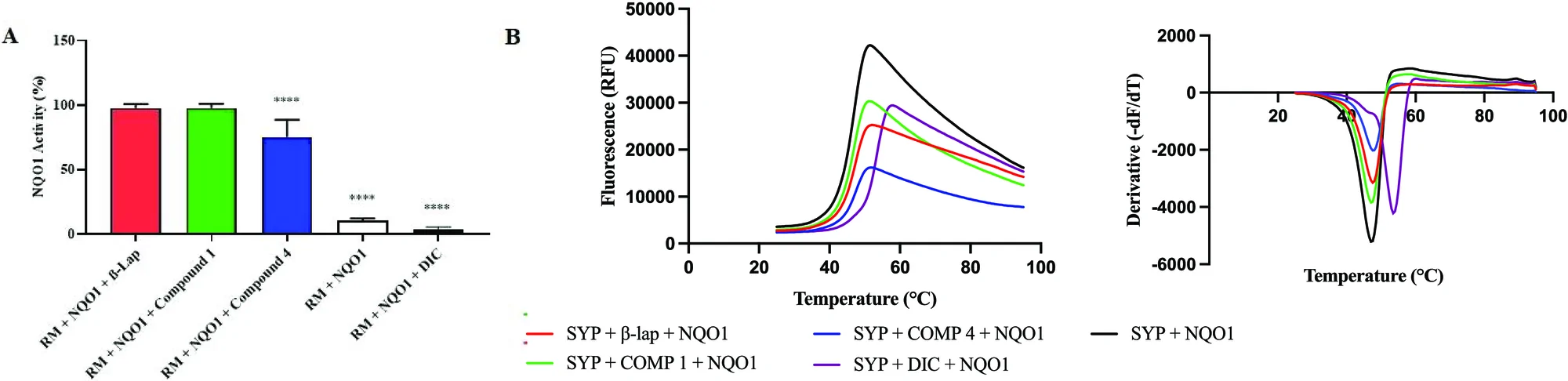
Enzymatic activity of NQO1 against **1** and **4.** (A) Relative enzymatic activity test and (B) thermal Shift assay, fluorescence emission profiles as a function of temperature of the NQO1 protein alone and its complexes with the ligands β-Lapachone (β-Lap), **1**, **4**, and dicoumarol (DIC) during thermal denaturation. Data are presented as mean ± SEM of three independent experiments conducted in triplicate. Mean comparison was performed using ANOVA followed by Tukey’s post-test (GraphPad Prism 8.0). Statistical significance was determined by comparing treatment group and β-Lapachone control, denoted as **** *p* < 0.0001. RM: Reaction Mixture, SYP: Sypro Orange dye.

### Molecular Docking

The results of molecular docking suggest that the naphthoquinones studied bind to the active site of NQO1 and likely interact with amino acid residues present in this site, such as Trp105, Phe178 (**1**), Trp105, Tyr126, Tyr128, Gly149, Gly150, Phe178, and His194 (**1** and **4**). Compared to **1**, compound **4** showed lower binding energy and, therefore, likely higher affinity for NQO1 (See ESI, Figure S3,).

### Quantum Biochemistry Simulations

Experimental evidence indicates that the molecule **4** interacts with the enzyme NQO1. Molecular docking simulations demonstrate that **4** binds to the same active site as Dicoumarol (DIC). To analyze the interactions between the amino acids of the NQO1 active site and **4** both qualitatively and quantitatively, we conducted quantum biochemistry calculations. Additionally, we applied the same methodology to assess the interaction of DIC with NQO1, using this data as a benchmark for comparison between the two ligands.

The results of distribution of interaction energies (Supporting Information) indicate that the MFCC model accurately represents the system. The interaction energy between the simulated ligands and the amino acids at the NQO1 binding site decreases with increasing distance, converging to zero around 14 Å, which defines the effective interaction range. Figure S4 also highlights the residues contributing most to the stabilization of ligand **4** and dicoumarol. Tyr128 (chain C) exhibits interaction energies of approximately −22 kcal·mol^–1^ with **4** and – 24 kcal·mol^–1^ with DIC, while Trp105 (chain C) contributes −20 kcal·mol^–1^ and −16 kcal·mol^–1^, respectively. Additional residues, including Phe178 and Pro68 (chain C) and Gln104 and Gly149 (chain A), display interaction energies above −10 kcal·mol^–1^ with **4**, reinforcing their role in complex stabilization.

The interaction energy profiles (Supporting Information) were obtained by summing the interaction energies of NQO1 residues within the same radius as each ligand. The profiles show that the interaction energy between the ligands (DIC and **4**) and NQO1 residues decreases sharply with decreasing distance, indicating stronger ligand–protein affinity at shorter ranges. Figure S1 also separates the interaction energies by chain, highlighting the amino acids from chains A and C responsible for ligand stabilization. Overall, **4** exhibits a stronger and more stable interaction with NQO1 (−164.9 kcal·mol^–1^) than DIC (−104.9 kcal·mol^–1^), evidencing its higher binding potential and energetic contribution to complex stability. Binding Site, Interaction Energy, and Residue Domain (BIRD) analysis (Figure S2) identifies the key residues in the A (pink) and C (orange) chains of the NQO1 protein that contribute to the stabilization of the DIC and **4** ligands. Residues Trp105, Phe106, Gly149, Gly150, His161, Met154, Tyr126, and Tyr128 in chain A, and Pro68, Phe178, and Phe236 in chain C, exhibit the highest energetic contributions to ligand binding. The short distances between ligand and residue atoms suggest the presence of strong noncovalent interactions. The 2D schematic representation highlights the types of interactions Pi–Pi and Pi–Pi-alkyl stacking, hydrogen bonding, and van der Waals interactions—and maps the specific ligand regions involved in binding. Overall, the analysis provides a comprehensive understanding of the molecular and energetic determinants responsible for stabilizing the DIC and **4** complexes with NQO1.

Expanding on previous findings that highlighted the antiproliferative properties of naphthoquinones in prostate cancer models, the present study systematically investigated the *in vitro* antitumor activity and underlying mechanisms of **4** and its parent compound **1** against the NQO1-overexpressing DU-145 prostatic adenocarcinoma cell line. To clarify the mechanistic basis of their cytotoxicity, the research combined computational approaches, including molecular docking, with experimental validation through enzymatic assays targeting NQO1, a key redox enzyme involved in quinone bioactivation. This integrated strategy aimed to elucidate structure–activity relationships, redox-dependent cytotoxic mechanisms, and pharmacodynamic differences between the mono- and dual-redox-center analogs, thereby establishing the dual-redox architecture as a promising chemical platform for antineoplastic drug design within the broader field of quinone-based agents.^15^

Several authors have studied naphthoquinones and their cytotoxic potential against human cancer cell lines.^16^ Notably, quinones incorporating two redox centers have been reported to exert enhanced cytotoxic activity against tumor cell lines compared with their monoredox-center parent molecules, while demonstrating reduced potency against specific nonmalignant cell lines.^13,17,18^ Naphthoquinones bearing two redox centers were synthesized and demonstrated potent cytotoxic activity against A549 cells (IC_50_ < 2.21 μM), alongside attenuated cytotoxicity toward the tested nontumorigenic cell lines (IC_50_ > 32.17 μM), suggesting the superior antiproliferative activity of the dual-center redox systems against cancer cells. Additionally, these compounds displayed enhanced affinity for the enzyme NQO1.^18^ Da Silva Júnior and Braga groups^12^ reported the synthesis of 47 new quinone-based structures joining two redox centers, several of these compounds presented IC_50_ values below 0.5 μM with good selectivity index, demonstrating their cytotoxic potential as new anticancer prototypes.^12^

Similarly, our results demonstrated the superior potency of compound **4**, a dual-redox molecule, compared with the monoredox architecture of compound **1**. Furthermore, compound **4** exhibited 2-fold higher cytotoxicity against DU-145 cells relative to nonmalignant human MRC-5 fibroblasts.^19^ Our data suggest that the incorporation of two quinonoid nuclei (i.e., two redox centers) exerts a synergistic effect, resulting in molecules with enhanced cytotoxic activity against tumorigenic cell lines.^13^ The cytotoxicity displayed by these compounds in DU-145 cells can be attributed, at least in part, to high levels of NQO1. This enzyme is frequently overexpressed in diverse malignancies and facilitates the bioactivation of naphthoquinones, as previously described for β-lapachone.^20^ These findings suggest that high NQO1 expression may increase cancer cell susceptibility to quinone-mediated redox cycling. Moreover, although prostate cancer cells were the primary focus of this study owing to the high global burden of the disease,^2^ the dual-quinone scaffold warrants future testing against a broader panel of cancer types to establish its potential as a general platform for discovering novel dual-redox anticancer agents.

However, the high levels of the enzyme NQO1 and other oxidoreductases are not exclusive to tumor cells. Healthy organs and tissues with elevated metabolic and detoxification rates, such as the liver and the renal tubular epithelium, express an abundance of phase I/II reductases^21a^ that could bioactivate compound **4** and trigger cytotoxicity, damaging nontumorigenic sites. Importantly, normal tissues, especially in the liver, typically possess robust endogenous antioxidant defense systems (e.g., high baseline reduced glutathione levels, catalase, and superoxide dismutase) that may help counteract, tolerate or buffer transient drug-induced oxidative stress^21b,21c^ . In contrast, extensive exposure of off-target organs to high systemic levels of oxidant-inducing drugs, or even drug accumulation in these sites, may exhaust endogenous antioxidant defenses^21d^. These concerns apply to dual-redox quinone scaffolds and must be addressed in future preclinical toxicology studies with compound **4** and its derivatives. For example, the potential organ toxicity could be attenuated using prodrug approaches, antibody-drug conjugation, organ-targeted delivery systems, controlled-dosing fractionation and scavenger coadministration to allow normal tissue recovery, while preserving cytotoxic action in target tumors.^21e–21g^

Morphological features of DU-145 cells treated with compounds **1** and **4** after 24 h of exposure, revealed that **4** caused an increase in cytoplasmic size, loss of membrane integrity, and vacuoles, indicative of cell death. Treatment with **1** revealed vacuoles, loss of membrane integrity, and apoptotic bodies, suggesting the initiation of an apoptotic process. The positive control (β-lapachone) induced chromatin condensation in the nucleus, cell shrinkage, and irregularities in cell shape, changes consistent with apoptotic cell death.^22^ These morphological phenotypes, combined with membrane integrity and cell density data, demonstrated that there was indeed an intense process of membrane integrity loss and a decrease in cell density, both concentration-dependent processes, corroborating the cytotoxicity of synthetic naphthoquinones.

Since many naphthoquinone derivatives are hemolytic agents,^23^ the hemolytic activity of **1** and **4** was investigated. The results for both molecules were considered low, with 2.58 ± 0.35% for **1** and 1.04 ± 0.20% for **4**. Therefore, we can discard the possibility of direct interaction of the molecules with the cell membranes, whether through rupture or pore formation, suggesting that more specific mechanisms are associated with the antiproliferative effects.

In the assessment of cell death mechanisms involved in the cytotoxicity, the assays demonstrated that deferoxamine (DFO), an iron chelator, inhibited the cytotoxic activity of **4** by more than 3–4 times, confirming that the presence of iron cations is crucial for the induction of cell death. This result strongly suggests the occurrence of ferroptosis in DU-145 cells treated with **4**, an iron-dependent cell death.^24^ In cells treated with **1**, there was a significant increase in cell viability when exposed to the caspase **3** inhibitor, considered an executioner caspase of apoptosis, due to its role in coordinating the destruction of cellular structures such as DNA fragmentation or degradation of cytoskeletal proteins.^25^ Cells treated with **1** also showed increased cell viability in the presence of higher concentrations of DFO, suggesting less intense occurrence of ferroptosis compared to cells treated with 4. The slight, nonsignificant increases with specific cell death inhibitors also suggest that apoptosis, necrosis, autophagy, and ferroptosis may contribute to the cell death induced by compound 1**1**.

Morphological features of ferroptosis may include loss of membrane integrity, increased cytoplasmic size, moderate chromatin condensation, loss of adherence to the substrate, rounding, as well as an increase in the number of phagosomes.^26c^ Morphological changes like these were observed in cells treated with the molecule **4**. Consistent with our observations,^27^ Caro and colleagues identified a synthetic naphthoquinone derivative that suppresses colorectal cancer progression via dual mechanisms of ferroptosis induction and suppression of the MAPK signaling cascade.^27^ Complementing these findings, Du et al.^28^ reported concentration-dependent cytotoxicity (IC_50_ ≈ 18.5 μM) for juglone, a natural naphthoquinone, in A549 human lung adenocarcinoma cells, mediated by concurrent activation of apoptotic and ferroptotic pathways, highlighting its potential as a therapeutically relevant multitarget agent.

One of the most well-known mechanisms of action of quinones is the production of ROS, which is also characteristic of cell death by ferroptosis.^26c^ To investigate whether cellular oxidative stress was involved in cytotoxicity mediated by compounds **1** and **4**, 5 mM NAC was used as a pretreatment. NAC pretreatment increased the IC_50_ values, indicating that it partially inhibited the cytotoxic activity of the compounds. Compound **4** was the most affected, since it exhibited an approximately 3-fold increase in its initial IC_50_ value, suggesting a potential correlation between its cytotoxicity and ROS production. Interestingly, molecule **1** did not exhibit a significant rise in IC_50_. Using fluorescence microscopy, it was observed that **4** significantly induced the production of ROS, particularly after 6 h of treatment, whereas molecule **1** did not induce ROS production significantly. Elevated ROS levels are known to be associated with mitochondrial depolarization. Indeed, mitochondria play a pivotal role in various cell death processes, such as apoptosis, necroptosis, pyroptosis, autophagy, mitophagy, and ferroptosis.^29^ Remarkably, both molecules caused significant mitochondrial depolarization at both treatment times, with compound **4** showing particularly high depolarization at 1 μM, specifically at the 6-h time point, the reduction in mitochondrial potential in the early hours of treatment is common, as mitochondria are involved in the initial stages of the cell death process.^14^ Lipid peroxidation is also a keypoint in ferroptosis, can be generally described as a process in which oxidants, such as free radicals, attack lipids containing carbon–carbon double bonds, especially polyunsaturated fatty acids.^30^ Using the C11-Bodipy sensor in the cells after 6 h of exposure to the compounds, we observed that lipid peroxidation was significantly higher in cells treated with compound **4**, a result consistent with the elevated levels of ROS obtained. Lipid peroxidation can disrupt membrane assembly, causing changes in fluidity and permeability, alterations in ion transport, and inhibition of metabolic processes. Injuries to mitochondria induced by lipid peroxidation can lead to increased ROS generation.^31^ In ferroptosis, the Fenton reaction plays a role, where iron contributes to ROS formation within cells, this amplifies phospholipid oxidation, intensifying lipid peroxidation and oxidative damage to cells.^26^ Such processes may be observed in DU-145 cells treated with **4**. Iron is the most prevalent metal within mitochondria, during ferroptosis, voltage-dependent anion channels (VDAC) open, and iron accumulates in mitochondria. VDAC permeability can be altered by drugs, causing oxidative damage, mitochondrial dysfunction, increased mitochondrial ROS levels, mitochondrial membrane potential depolarization, mitochondrial swelling, and subsequent oxidative cell death.^32^

DNA damage is a quite common mechanism of action of quinones.^33^ Therefore, DNA damage was verified by the flow cytometric DNA fragmentation and the comet assays after a 24-h treatment with the naphthoquinones. DNA Fragmentation was evident and significant in treatment with both compounds, observing a concentration and time-dependent process. When cells were treated with NAC 5 mM (an antioxidant agent) before exposure to naphthoquinones, there was a statistically significant decrease in DNA fragmentation, suggesting the contribution of ROS to DNA damage.^34^ Sequentially, comet assay was performed, as there are two main versions of the comet assay, the neutral version (pH 7.0–8.5) was mainly used to detect double-strand DNA breaks^35^ and the alkaline version (pH > 13.0) increased the assay’s sensitivity to detect smaller amounts of DNA damage, including single-strand and double-strand DNA breaks, alkali-labile sites, incomplete excision repair sites, and cross-links.^36^ In the comet assay, reinforcing the involvement of free radicals in DNA damage induction, pretreating with NAC effectively mitigated the genotoxic effects of both **1** and **4**. Additionally, the coincubation with the hOGG1 enzyme notably increased the number of DNA strand breaks induced by these compounds. The enzyme identifies oxidative DNA damage by recognizing and cleaving affected bases. These results align with the established notion that free radicals, including ROS, play a pivotal role in initiating DNA double-strand breaks and trigger cell death in cancer cells.^34^ Notably, compound **4** demonstrated a predominant induction of this type of DNA break, confirming the involvement of oxidative stress in DNA damage.^37^ In this context, Da Silva et al. (2022)^38^ showed the antitumoral effect of a synthetic naphthoquinone via ROS-mediated DNA damage and apoptosis in colon cancer cell line.^38^

Since quinones are known to induce ROS in redox reactions, with the possible involvement of the enzyme NQO1, the interaction between **1** and **4** with the enzyme was investigated using *in silico* geometric docking. The binding site for compounds with high affinity for the enzyme, such as dicumarol, includes residues Trp105, Phe106, Tyr126, Tyr128, Met131, Gly149, Gly150, Met154, Tyr155, His161, Gly174, Phe178, His194, Phe236, and FAD-residue301.^39^ **1** and **4** likely interact with amino acid residues at these binding sites, with potential interactions shared with residues Trp105, Phe178 for **1** and Trp105, Tyr126, Tyr128, Gly149, Gly150, Phe178, and His194 for **4**. Among the two molecules, **4** stands out for exhibiting the lowest RMSD value and lower binding energy. Lower binding energy values indicate greater stability of the complex, and favorable RMSD values reaffirm the viability of the ligand–receptor complex formation.

To investigate the interactions between the amino acids of the NQO1 active site and **4**, both qualitatively and quantitatively, we performed quantum biochemistry calculations. In addition, we applied the same methodology to evaluate the interaction of DIC with NQO1, using these data as a reference for comparison between the two ligands. From the results obtained from the quantum simulations using the MFCC methodology and applying an average dielectric constant of 40, we calculated all the interaction energies between the residues of the NQO1 binding site and the ligands DIC and **4**. We can see that the most energetically significant amino acids are located near the ligands, with the closest distance being around 2.0 Å. In contrast, the most distant residues are about 40 Å away and exhibit interaction energies near zero. This observation confirms that the quantum biochemistry calculations employed accurately represent the expected behavior of the interaction energy between the residues and the ligands.

The interaction energy versus distance indicates that ligand **4** exhibits a stronger interaction with the NQO1 protein compared to dicoumarol, further corroborating the findings from the docking simulations. Analysis reveals that chain A of NQO1 contributes an interaction energy of −47.9 kcal·mol^–1^, while chain C contributes −57.0 kcal·mol^–1^. For the ligand identified as **4**, chain A exhibits an interaction energy of −87.2 kcal·mol^–1^, and chain C shows −77.6 kcal·mol^–1^. The calculated total interaction energy for the ligands with the protein, which is the aggregate of the interaction energies from chains A and C, amounts to −104.9 kcal·mol^–1^ for DIC and −164.9 kcal·mol^–1^ for **4**. Additionally, the results provide information on which amino acids contribute to the stabilization of each ligand and to which chain of the protein they are associated. In the case of **4** (docking data), we can observe that chain A plays a more significant role in stabilizing the complex than chain C. The amino acids in chain C that contribute most to the stabilization of 4-NQO1 complex are Pro68, Phe178, Tyr67, Tyr128, and Tyr126. Regarding chain A, we can highlight Trp105, Trp106, Pro102, Met154, Gln104, and Gly149. For the DIC-NQO1 complex, we can highlight the amino acids that contribute most energetically to the stabilization of dicoumarol, which belong to the C chain and include Phe236, Tyr128, Tyr126, Phe178, and Met131. In contrast, for the A chain, we can highlight Phe106, Gly149, Gly150, His161, and Trp105 as the most significant.

The BIRD analysis indicated that the molecular structures of DIC and **4** interact with the amino acids in the binding site of the NQO1 protein in distinct ways. Specifically, **4** can engage with a greater number of amino acids than dicoumarol, owing to its molecular structure featuring more interaction regions. Notably, we can identify certain amino acids that have no interaction with dicoumarol but interact more significantly with **4**. We also highlight the amino acid Tyr128, which has a strong interaction energy with both DIC and **4**, with values close to −24 kcal·mol^–1^ for DIC and −22 kcal·mol^–1^ for **4**. Asher and collaborators have already highlighted that the amino acid Tyr128 plays a crucial role in the interaction and stability of dicoumarol in NQO1.^39^ Through our results, we showed that this residue also plays an essential role in the stabilization of **4**. Furthermore, Asher et al.^39^ reported that other amino acids, such as Phe232, His161, Gly150, and Tyr126, are also important for the stabilization of DIC. Still, we observed in BIRD that the residues have a moderate interaction energy (around −8 kcal·mol^–1^) for both DIC and **4**, and Phe232 did not present a significant interaction energy. Our results reinforce the need to use quantum calculations to describe ligand-protein interactions with greater precision and detail, thereby offering more reliable evidence for the development of new NQO1 ligands.

Several authors have reported that quinones can act as substrates of NQO1.^40,41^ Since the DU-145 cell line exhibits high NQO1 expression, a phenotype shared across various malignant tumors, cytotoxicity assays were performed in the presence and absence of dicoumarol, a competitive NQO1 inhibitor.^39^ The result showed that there was a decrease in the cytotoxic activity of the molecules in the presence of dicoumarol, proving that the enzyme is important for the antiproliferative mechanism, mainly for **4** and for β-lapachone, that has been confirmed to selectively increase ROS levels in cancer cells in a NQO1-dependent manner. However, in the HL-60 cell line, compounds **1** and **4** also exhibited high cytotoxic potential and the presence of the inhibitor did not affect significatively the IC_50_, which may be explained by the likely existence of other enzymes that could be involved in the process of bioactivation or reduction of quinones, proving **4** is not completely dependent on NQO1. Therefore, although NQO1 may contribute to the mechanism of action of synthetic naphthoquinones, it is not essential for their cytotoxic activity.

The assessment of relative enzymatic activity using the human recombinant NQO1 revealed that the compounds acted as substrates for the enzyme, like β-lapachone. Interestingly, **4** exhibited the higher cytotoxicity, but the lowest relative enzymatic activity compared to β-lapachone or **1**. The results demonstrate that unlike β-lapachone, a molecule activated by NQO1,^42^ **4** is not fully dependent on this enzyme for its cytotoxic effect, being probably and even preferentially reduced via one-electron reaction, catalyzed by enzymes other than NQO1, yielding less stable semiquinones and free radicals. This could explain the high levels of ROS generated by the naphthoquinone with two redox centers (**4**).

In cellular redox cycles, quinones undergo enzyme-catalyzed one-electron reduction (e.g., mediated by liver-abundant cytochrome P450 reductases that drive phase I drug metabolism and prodrug activation). The resulting semiquinones are rapidly oxidized by molecular oxygen to regenerate the parent quinone. This futile cycling depletes reducing equivalents and generates reactive oxygen species (ROS), causing indiscriminate cellular damage until the quinones or semiquinones are neutralized. Alternatively, NQO1-catalyzed two-electron reduction yields stable hydroquinones, preventing ROS generation. Under these conditions, NQO1 acts as an antioxidant defense, neutralizing anticancer quinones and promoting chemoresistance. However, depending on the structure of the parent quinone, the derived hydroquinone can be unstable, reactive, and ROS-generating; in this scenario, NQO1 acts as an activator, promoting oxidative stress and anticancer activity.^43^

This latter activation pathway may apply to compounds **1** and **4**, which are NQO1 substrates characterized in this study. Indeed, NQO1 inhibition halved the potency of both compounds against DU-145 cells. This occurred even though the dual-redox-center compound **4** induced lower enzymatic activity than the single-center compound **1**, suggesting that **4** is less susceptible to NQO1 activation. Because both compounds exhibited potent cytotoxicity against NQO1-deficient HL-60 cells regardless of NQO1 inhibition, they are also activated by one-electron reductases. Thus, the activation of both compounds is only partially driven by NQO1, as this role is shared with other intracellular reductases.

Via either pathway, compounds **1** and **4** generate DNA-damaging ROS, triggering cell death. Unlike healthy cells, cancer cells exhibit high basal oxidative stress and limited reducing reserves, making them highly sensitive to further ROS elevation.^43c^ Accordingly, compounds **1** and **4** showed superior potency against neoplastic cells compared to nontumor human fibroblasts. Furthermore, robust ROS induction coupled with active ferroptotic machinery can trigger ferroptosis, as observed for compound **4**, in contrast to the apoptosis stimulated by compound **1**. Consequently, ferroptosis is probably favored by the intense ROS generation mediated by compound **4** within a cellular environment (DU-145 cells) enriched with abundant labile iron pool (Fe^2+^) and long-chain polyunsaturated fatty acids, alongside depleted or defective antiferroptotic proteins.^43^

In sequence, the thermal shift assay showed that only dicoumarol and β-lapachone were able to increase the melting temperature of their complexes with NQO1, compared to the isolated enzyme, this relates to the fact that these compounds have high affinity by the enzyme, dicoumarol being a competitive inhibitor with noncovalent bonds,^44^ and β-lapachone as a substrate, being a bioactivatable drug dependent on NQO1.^45^ In contrast, the melting temperature (Tm) of NQO1 alone was not statistically different from the Tm of the same enzyme in the presence of **1** and **4**. Therefore, the complexes of NQO1 with dicoumarol and β-lapachone are more stable than the complexes of this enzyme with the studied compounds. This result recapitulates the possible relative enzymatic activity of NQO1 in reducing **4**. Although NQO1 overexpression is a recognized therapeutic vulnerability in cancer, the clinical use of strictly NQO1-dependent quinones may be limited by the NQO1*2 polymorphism, which results in an unstable and catalytically inactive enzyme and has been associated with reduced responsiveness to agents such as β-lapachone. Importantly, compound 4 displayed only partial NQO1 dependence, retaining cytotoxic activity under NQO1 inhibition and in cells with lower NQO1 expression. This suggests that alternative reductive pathways may contribute to its activation, potentially reducing susceptibility to resistance mechanisms linked to NQO1 polymorphisms.

Thus, the proposed mechanisms of action indicate that compound **1** induces DNA damage and trigger apoptosis; conversely, compound **4** promotes ROS accumulation, culminating in oxidative stress, lipid peroxidation, DNA damage, and ultimately ferroptosis (Figure S5, ESI). Further investigation into the dual-redox system, as embodied by compound **4**, may reveal its utility against a wider spectrum of malignancies.

## Conclusions

The synthetic naphthoquinones demonstrated potent cytotoxicity toward DU-145 prostate cancer cells, chosen as a study model due to the high epidemiological burden of the disease. The dual-redox derivative compound **4** exhibited significantly greater antiproliferative activity compared with monoredox compound **1**. These findings underscore the anticancer potential of redox-active quinones against high-burden malignancies such as prostate cancer, encouraging further evaluation in additional cancer models. Mechanistic investigations revealed that compound **4** primarily triggers ferroptosis, whereas compound **1** induces apoptosis, both pathways involving partial NQO1-mediated redox cycling and oxidative stress. Consequently, compound **4** represents a promising lead candidate for broad-spectrum cancer screening, subsequent *in vivo* studies and drug discovery.

## Supplementary Material



## Notes

General remarks; substrate synthesis; biological assays: cell culture; cytotoxicity analysis; MTT Assay; hemolytic assay; assessment of the cell death mechanism; trypan blue exclusion; morphological examination of cell by light microscopy; flow cytometry analysis; evaluation of intracellular ROS; evaluation of lipid peroxidation; analysis of DNA strand breaks (Comet assay); investigation of the involvement of the NQO1 enzyme: MTT Assay; relative enzymatic activity; thermal Shift Assay; molecular docking; and quantum biochemistry simulations; statistical analysis; ^1^H and ^13^C NMR Spectra (PDF)
