# Supplementary material for: Prodrug-like Quinone Containing Two Redox-Active Centers Induces Ferroptosis in Prostate Cancer Cells with Cytotoxic Action Partially Depending on NAD(P)H Quinone Dehydrogenase 1 (NQO1)
Source: ACS Omega. 2026 Aug 25;11(35):52411–26. doi: 10.1021/acsomega.5c11250 (PMC13564003; doi:10.1021/acsomega.5c11250)
Supplement: Supplementary file 1 [file ao5c11250_si_001.pdf]

Supplementary Information

Prodrug-like quinone containing two redox-active centers induces ferroptosis in prostate cancer cells with cytotoxic action partially depending on NAD(P)H Quinone Dehydrogenase 1 (NQO1)

Eleicy N. Mendoza,[a] Maria Fernanda Madrid,[a] Pedro M. S. da Costa,[a] Serena Castelli,[b] Fabio Ciccarone,[c] Maria R. Ciriolo,[c] Bruno C. Cavalcanti,[a] Emmanuel S. Marinho,[d] Jacilene Silva,[d] Eveline M. Bezerra,[e] Roner F. da Costa,[e] Renata G. Almeida,[f] Joyce C. de Oliveira,[f] Francisco Washington A. Barros-Nepomuceno,[g] Manoel O. de Moraes Filho[a], Eufrânio N. da Silva Júnior[f]*, Claudia Pessoa[a]* and Carlos R. K. Paier[a]*

[a] Department of Physiology and Pharmacology, Drug Research and Development Center, Federal University of Ceara, Fortaleza, 60.430-275, Ceara, Brazil. Email: cpessoa@ufc.br; [b] Department for the Promotion of Human Science and Quality of Life, San Raffaele Open University, Via di Val Cannuta, 247, 00166, Rome, Italy; [c] Department of Biology, University of Rome “Tor Vergata”, Via della Ricerca Scientifica, 00133, Rome, Italy; [d] Laboratory of Bioprospecting and Monitoring of Natural Resources (LBMRN), Center of Science and Technology, State University of Ceará, Fortaleza, 60.714-903, Ceará, Brazil; [e] Federal Rural University of the Semi-Arid Region (UFERSA), Mossoró, 59625-900, Rio Grande do Norte, Brazil; [f] Department of Chemistry, Institute of Exact Sciences, Federal University of Minas Gerais, Belo Horizonte, 31270-901, Minas Gerais, Brazil. Email: eufranio@ufmg.br; [g] Institute of Health Sciences, University for International Integration of the Afro-Brazilian Lusophony (UNILAB), Redenção, 62790-000, Ceará, Brazil.

Table of Contents

General Remarks S1
Substrate Synthesis S1
Biological assays S4
Molecular Docking S10
^{1}H and ^{13}C NMR Spectra S17

General Remarks

The solvents were dried using molecular sieves in inert atmosphere storage. Lawsone and Juglone, were used as purchased without further purification. The reaction concentration is expressed in molar (M); this concentration was calculated by the ratio of the amount of the main reactant (the limiting agent) in mmol and the volume of the solvent applied in mL. The presented yields refer to isolated compounds, estimated to be >95% pure as determined by ^{1}H-NMR. TLC: Merck, TLC Silica gel 60 F254, detection at 254 nm. Infrared spectra were recorded on a Bruker ATR FT-IR Alpha device and IR Prestige-21 Shimadzu using KBr plates. Mass-spectra: EI-MS: Jeol AccuTOF at 70 eV; ESI-MS: Bruker maXis and MicrOTOF. High-resolution mass spectrometry (HRMS): Bruker maXis, Bruker MicrOTOF and Jeol AccuTOF. Melting points: Büchi 540 capillary melting point apparatus; values are uncorrected. The NMR spectra were recorded on Avance III HD 400, Avance III 400, and Avance NEO 600 instruments. If not otherwise specified, chemical shifts (δ) are provided in ppm. ^{13}C-NMR shifts are classified as: Cq (non-hydrogenated carbon), CH, CH_2, and CH_3, indicating the nature of the carbon assigned, according to what was observed by DEPT or ATP analysis. All of the structure names were given under IUPAC rules by the CS ChemDraw Ultra program.

Substrate Synthesis

3-azido-2,2-dimethyl-2,3-dihydronaphtho[1,2-*b*]furan-4,5-dione (1):

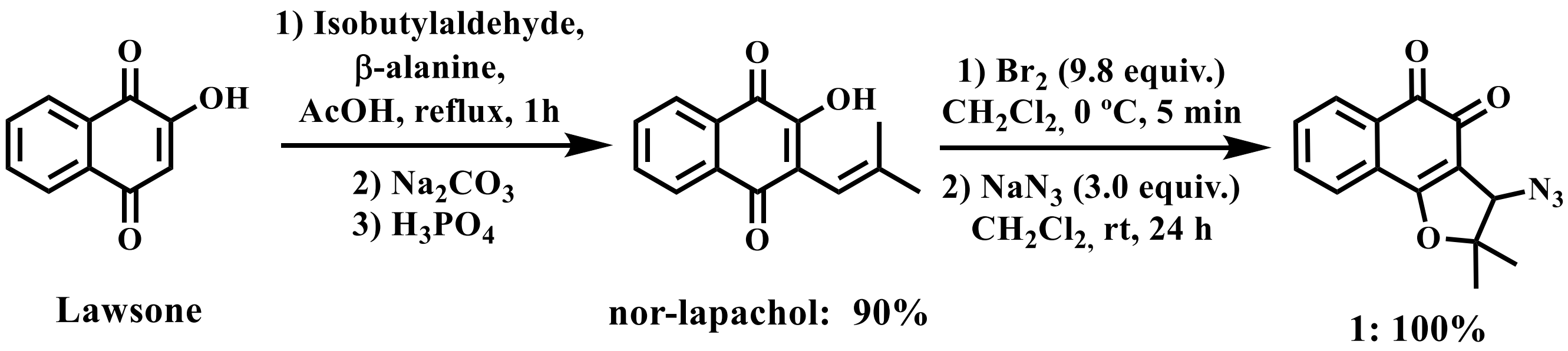

Synthesis of 3-azido-2,2-dimethyl-2,3-dihydronaphtho[1,2-*b*]furan-4,5-dione (1): In a 100mL rounded bottom flask, nor-lapachol (456 mg, 2.0 mmol), previously prepared as described in the literature,[1] and DCM (30 mL) were added. The mixture was cooled down to 0 ºC, followed by the careful addition of bromine (1.0 mL, 3.12 g, 19.5 mmol). The reaction was kept under continuous stirring at 0 ºC for 5 min. The excess bromine, along with the solvent, was removed under reduced pressure, resulting in an orange

[1] David, C. C.; Lins, A. C. S.; Silva, T. M. S.; Campos, J. F.; Silva, T. G.; Militão, G. C. G.; Camara, C. A. Synthesis and cytotoxicity evaluation of a series of 3-alkenyl-2-hydroxy-1,4-naphthoquinones obtained by an efficient Knoevenagel condensation. *J. Braz. Chem. Soc.* **2019**, *30*(1), 8-18.

solid. This mixture was directly used without further purification in the next step through the addition of DCM (10 mL) and sodium azide (390 mg 6.0 mmol). The reaction was kept under continuous stirring at room temperature for 24 h. The final crude was suspended in 15 mL of distilled water, extracted with ethyl acetate (3 x 15 mL), and dried over Na_2SO_4. Column chromatography (*n*-hexane/AcOEt 8:2) on silica gel led to the desired azide (**1**) (538 mg, 100%) as an orange solid. ^{1}H NMR (400MHz, $CDCl_3$): 8.15–8.13 (m, 1H), 7.72–7.65 (m, 3H), 4.78 (s, 1H), 1.68 (s, 3H), 1.56 (s, 3H). ^{13}C NMR (100MHz, $CDCl_3$): 180.6, 175.5, 170.4, 134.8, 133.1, 131.4, 129.9, 127.0, 125.4, 113.8, 95.8, 67.7, 27.4, 22.2. IR (KBr): 3417, 2965, 2935, 2110, 1697, 1654, 1618,1571, 1406, 1266, 1217 cm^{-1}. m.p. (°C) = 200–202. The analytical data are in accordance with those reported in the literature.[2]

Note: *Diazidomethane can be generated from sodium azide in the presence of dichloromethane. Low-molecular-weight azides may undergo unpredictable decomposition, requiring particular caution during handling. Therefore, for larger-scale syntheses of azido-quinone derivatives, the use of dichloromethane should be avoided, and alternative solvents (e.g., DMF) are recommended to minimize potential safety risks.*[3]

5-benzyloxy-1,4-naphthoquinone (2) and 8-benzyloxy-2-(prop-2-yn-1-ylamino)naphthalene-1,4-dione (3):

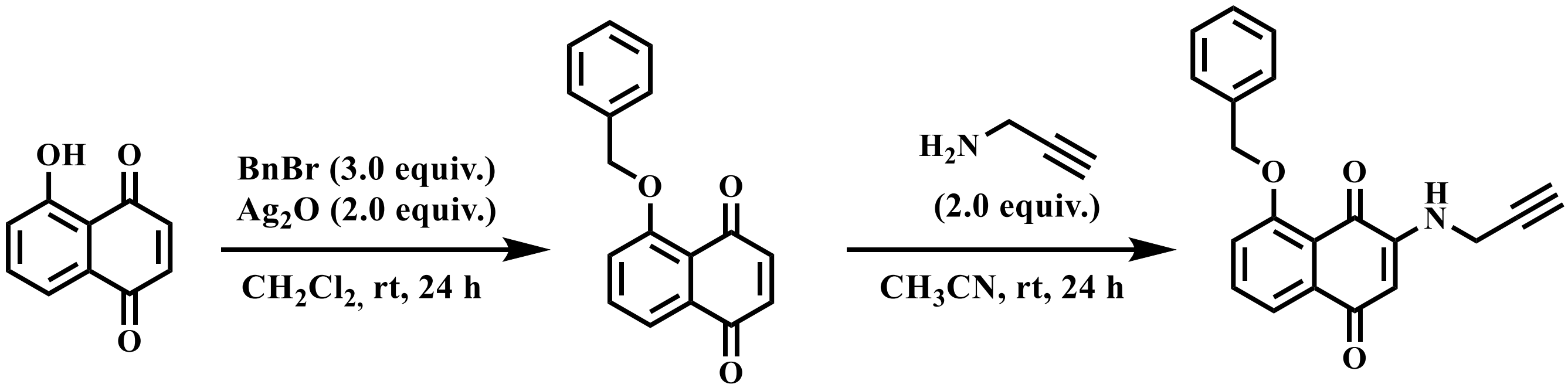

Juglone **2: 52%** **3: 49%**

Synthesis of 5-Benzyloxy-1,4-naphthoquinone (2): Juglone (174 mg, 1.0 mmol), benzyl bromide (513 mg, 3.0 mmol), and Ag_2O (463 mg, 2.0 mmol) were dissolved in dichloromethane (30 mL) in a 125 mL rounded-bottom flask. The solution was kept under stirring at 25 °C for 24 h. The final solution was filtered through a pad of celite and washed with dichloromethane. The solution was then concentrated under reduced pressure and purified through column chromatography (*n*-hexane/AcOEt 8:2) to provide 5-benzyloxy-1,4-naphthoquinone (**2**) (137 mg, 52%) as a red solid. ^{1}H NMR (400 MHz, $CDCl_3$): 7.71 (dd, *J*

---

2. da Silva Júnior, E. N.; Menna-Barreto, R. F. S.; Pinto, M. C. F. R.; Silva, R. S. F.; Teixeira, D. V.; de Souza, M. C. B. V.; de Simone, C. A.; de Castro, S. L.; Ferreira, V. F.; Pinto, A. V. Naphthoquinoidal [1,2,3]-Triazole, a New Structural Moiety Active against Trypanosoma cruzi. *Eur. J. Med. Chem.* **2008**, *43*, 1774–1780.

3 Bräse, S.; Gil, C.; Knepper, K.; Zimmermann, V. Organic Azides: An Exploding Diversity of a Unique Class of Compounds. *Angew. Chem. Int. Ed.* **2005**, *44*, 5188–5240.

= 7.6, 0.8 Hz, 1H), 7.62 (t, *J* = 8.4 Hz, 1H), 7.57 (d, *J* = 7.2 Hz, 2H), 7.40 (t, *J* = 7.2 Hz, 2H), 7.33–7.31 (m, 2H), 6.86, (s, 2H), 5.27 (s, 2H). ^{13}C NMR (100 MHz, $CDCl_3$): 185.3, 184.2, 158.6, 140.9, 136.3, 136.1, 134.9, 134.2, 128.8, 128.0, 126.7, 120.3, 119.7, 119.5, 70.9. IR (KBr): 1747, 1660, 1614, 1582, 1497, 1454, 1254, 1023, 733, 697 cm^{-1}. m.p. (°C) = 30–32; HRMS (ESI): Calcd. for $C_{17}H_{12}O_3Na$ $[M+Na]^+$ 287.0679, found 287.0677. The analytical data are in accordance with those reported in the literature.[4]

Synthesis of 8-benzyloxy-2-(prop-2-yn-1-ylamino)naphthalene-1,4-dione (3): The 5-benzyloxy-1,4-naphthoquinone (**2**) (264 mg, 1.0 mmol) was dissolved in acetonitrile (3.0 mL) at room temperature in a 10 mL rounded-bottom flask. *N*-propargylamine (128 µL, 110.2 mg, 2.0 mmol) was added to the mixture and it was kept under continuous stirring over 24 h at room temperature. Purification by column chromatography on silica gel (*n*-hexane/EtOAc 8:2) yielded 8-benzyloxy-2-(prop-2-yn-1-ylamino)naphthalene-1,4-dione (**3**) (155mg, 49%) as a brown solid. ^{1}H NMR (400MHz, DMSO-d_6): 7.82 (t, *J* = 6.0 Hz, 1H), 7.76 (t, *J* = 8.0 Hz, 1H), 7.61–7.59 (m, 3H), 7.50 (d, *J* = 8.4 Hz, 1H), 7.41 (t, *J* = 7.2 Hz, 2H), 7.33 (t, *J* = 7.6 Hz, 1H), 5.69 (s, 1H), 5.30 (s, 2H), 4.01 (dd, *J* = 5.6, 2.0 Hz, 2H), 3.23 (t, *J* = 2.0 Hz, 1H). ^{13}C NMR (100 MHz, DMSO-d_6): 181.3, 179.5, 158.6, 149.0, 136.7, 136.1, 135.2, 128.4, 127.7, 127.0, 118.2, 118.2, 118.2, 99.8, 79.1, 74.5, 70.1, 31.2. IR (KBr): 3359, 3289, 2359, 1674, 1601, 1577, 1494, 1252, cm^{-1}. m.p. (°C) = 50–55. The analytical data are in accordance with those reported in the literature.[4, 5]

3-(4-(((8-benzyloxy-1,4-dioxo-1,4-dihydronaphthalen-2-yl)amino)methyl)-1*H*-1,2,3-triazol-1-yl)-2,2-dimethyl-2,3-dihydronaphtho[1,2-*b*]furan-4,5-dione (4):

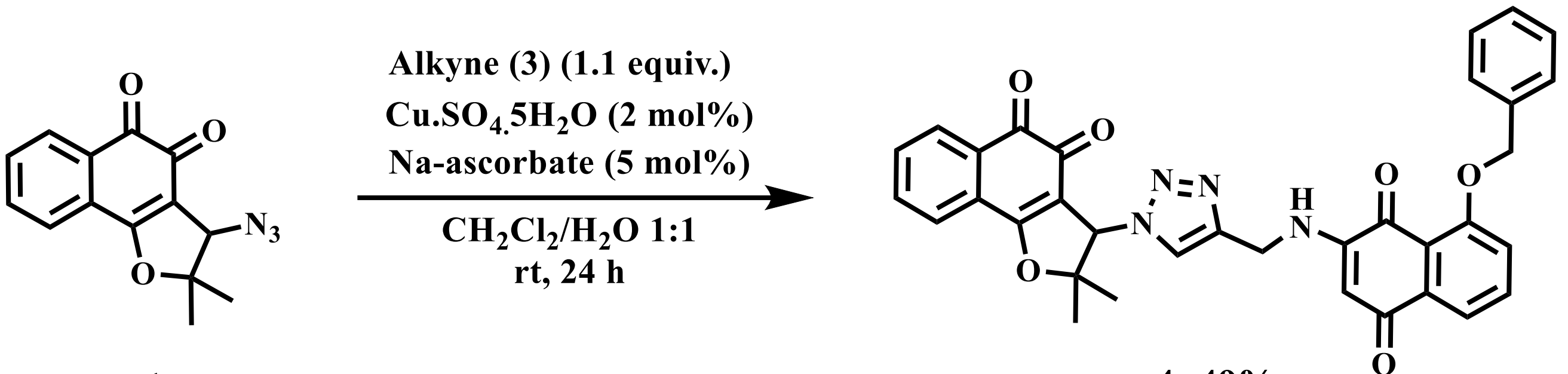

1 **4: 49%**

Synthesis of 3-(4-(((8-benzyloxy-1,4-dioxo-1,4-dihydronaphthalen-2-yl)amino)methyl)-1*H*-1,2,3-triazol-1-yl)-2,2-dimethyl-2,3-dihydronaphtho[1,2-*b*]furan-4,5-dione (4): A reaction tube was charged with the corresponding azide-lapachone derivative (**1**) (54 mg, 0.2 mmol, 1.0 equiv.), amino-alkyne

---

4. Mezeiova, E.; Spilovska, K.; Nepovimova, E.; Gorecki, L.; Soukup, O.; Dolezal, R.; Korabecny, J.; Kuca, K.; Sepsova, V. 2-Propargylamino-Naphthoquinone Derivatives as Multipotent Agents for the Treatment of Alzheimer's Disease. *Eur. J. Med. Chem.* **2021**, *211*, 113112.

5. Mezeiova, E.; Spilovska, K.; Nepovimova, E.; Gorecki, L.; Soukup, O.; Dolezal, R.; Korabecny, J.; Kuca, K.; Sepsova, V. 2-Propargylamino-Naphthoquinone Derivatives as Multipotent Agents for the Treatment of Alzheimer's Disease. *Eur. J. Med. Chem.* **2021**, *211*, 113112.

naphthoquinone (**3**) (70 mg, 0.22 mmol, 1.1 equiv.), $CuSO_4.5H_2O$ (1 mg, 0.002 mmol, 1 mol %), and sodium ascorbate (4 mg, 0.01 mmol, 5 mol %). Then, a mixture DCM/distilled water 1:1 (4 mL) was added. The reaction mixture was kept under vigorous stirring at room temperature for 24 h. The crude product was partitioned with distilled water (20 mL) and DCM (3 x 30 mL). Purification by column chromatography on silica gel (n-hexane/EtOAc 4:1) yielded (**4**) (57 mg, 49%) as an orange solid. ^{1}H NMR (600 MHz, DMSO-d_6): 8.25 (s, 1H), 8.04 (d, *J* = 7.2 Hz, 1H), 7.92 (t, *J* = 6.0 Hz, 1H), 7.85–7.74 (m, 4H), 7.60 (d, *J* = 7.2 Hz, 2H), 7.57 (d, *J* = 7.8 Hz, 1H), 7.50 (d, *J* = 8.4 Hz, 1H), 7.41 (t, *J* = 7.2 Hz, 2H), 7.33 (t, *J* = 7.2 Hz, 1H), 6.02 (s, 1H), 5.62 (s, 1H), 5.30 (s, 2H), 4.44 (d, *J* = 5.4 Hz, 2H), 1.67 (s, 3H), 0.98 (s, 3H). ^{13}C NMR (150 MHz, DMSO-d_6) 181.0, 179.8, 179.6, 174.6, 169.9, 158.6, 149.2, 143.2, 136.7, 136.1, 135.3, 134.8, 133.1, 131.8, 128.8, 128.4, 127.7, 127.0, 126.6, 125.1, 123.5, 118.2, 118.1, 111.3, 99.0, 95.3, 70.1, 66.1, 40.1, 37.6, 27.0, 20.6. IR (KBr): 3372, 2362, 1651, 1609, 1571, 1497, 1356, 1279, 1053 cm^{-1}. m.p. (°C) = 205–210. HRMS (ESI): Calcd. for $C_{34}H_{27}N_4O_6$ $[M+H]^+$ 587.1931, found 587.1929. The analytical data are in accordance with those reported in the literature.[6]

Biological assays

Cell culture

Human cancer cell lines (DU-145, prostate carcinoma; and HL-60, promyeoloblast cells) were obtained from the National Cancer Institute, Bethesda, MD, USA and human fibroblast cell line (MRC-5) was obtained from Cell Bank Rio de Janeiro, RJ, Brazil. The cell lines were maintained in RPMI-1640 medium (Gibco, Fisher Scientific™) supplemented with 10% fetal bovine serum (FBS) (Cultilab, Brazil), 100 U/mL penicillin, and 100 µg/mL streptomycin (Gibco, Fisher Scientific™), at 37 ºC and 5% CO_2 (NuAire™, USA). The non-tumoral cell lines were maintained in DMEM (Gibco, Fisher Scientific™) supplemented as previously described. Due to the epidemiological importance of prostate cancer, neoplastic cell lines originating from this tumor type, DU-145 was selected as the primary model due to its higher NQO1 expression compared to other prostate cancer cell lines (PC-3 and LNCaP), allowing direct evaluation of the proposed enzyme-dependent mechanism (Planchon et al., 2001). Preliminary experiments in PC-3 cells, which exhibit lower NQO1 levels, showed higher IC_{50} values for both compounds (compound 1, 6.511 ± 0.649 µM; compound 4, 1.139 ± 0.239 µM, after 72h of exposure), supporting partial NQO1 dependence. Therefore, DU-145 was selected for detailed mechanistic studies.

Cytotoxicity analysis

MTT Assay

[6] Oliveira, J. C.; de Carvalho, R. L.; Sampaio, H. G. S.; Honorato, J.; Ellena, J. A.; Martins, F. T.; Pereira, J. V. M.; Costa, P. M. S.; Pessoa, C.; Ferreira, R. S.; Araújo, M. H.; Jacob, C.; da Silva Júnior, E. N. It Takes Two to Tango, Part II: Synthesis of A-Ring Functionalised Quinones Containing Two Redox-Active Centres with Antitumour Activities. *Molecules*, **2023**, *28*, 2222.

The cytotoxicity of the compounds was assessed using the MTT assay, quantified by the ability of living cells to reduce the yellow dye 3-(4,5-dimethyl-2-thiozolyl)-2,5-diphenyl-tetrazolium bromide (MTT) to a purple formazan product.[7] Cells were seeded in 96-well plates (0.5-0.7 x 105 cells/mL) and treated with naphthoquinones dissolved in DMSO for 24, 48, or 72 hours. After incubation, the supernatant was withdrawn carefully and replaced with fresh medium containing MTT (0.5 mg/mL). Three hours later, the MTT's formazan product was dissolved in 100 µL of pure DMSO, therefore, the absorbance was measured at 595 nm (VICTOR Nivo™, Multimode Plate Reader, PerkinElmer Life and Analytical Sciences Ltd, Pontyclun, UK). Naphthoquinones were tested in concentrations ranging from 0.04 to 12.5 µM to determine the half-maximal inhibitory concentration (IC_{50}) from three independent experiments performed in triplicate. Stock solutions were prepared at a concentration of 5 mM. The control group received the same amount of vehicle. β-lapachone (Thermo Scientific, USA) was used a positive control. Final concentrations of DMSO in the culture media were kept below 0.4%. All prepared stocks are homogenized by vortexing, protected from light, and stored at 4 °C.

To assess the role of reactive oxygen species (ROS) in the cytotoxicity of the test compounds, cells were pre-treated with N-acetyl-cysteine (NAC) (5 mM) for four hours to restore intracellular reduced glutathione (GSH) levels and protect against oxidative stress.[8] After NAC treatment, the medium was removed, and cells were treated with naphthoquinones, a positive control, or vehicle, followed by incubation and viability assessment via the MTT assay.

For the initial evaluation of the mechanism of cell death, cells were pre-treated for two hours and co-treated with specific inhibitors, Z-VAD-FMK 25 µM (Sigma™, USA), deferoxamine (DFO) 50 µM and 100 µM (Sigma™, USA), necrostatin-1 100 µM (Sigma™, USA), 3-methyladenine (3-MA) 5 mM (Sigma™, USA), and caspase 3 inhibitor 25 µM (Calbiochem, US). Doxorubicin 3 µM (Sigma™, USA) was used as positive control, selected based on its extensively well-known and researched mechanism of action. **1** (4 µM) and **4** (1 µM) for 24 h were tested using the IC_{50} and incubated for 24 h. The plates were then incubated, and cell viability was assessed by the MTT assay, as previously described.

Hemolytic Assay

Mouse red blood cells are used due to their high similarity to human red blood cells, particularly in terms of sensitivity.[9] This experiment was submitted to and approved by the Ethics Committee on Animal Use at

7. Mosmann, T. Rapid Colorimetric Assay for Cellular Growth and Survival: Application to Proliferation and Cytotoxicity Assays. *J. Immunol. Methods.* **1983**, *65*, 55–63.
8. Sunilkumar, D.; Drishya, G.; Chandrasekharan, A.; Shaji, S. K.; Bose, C.; Jossart, J.; Perry, J. J. P.; Mishra, N.; Kumar, G. B.; Nair, B. G. Oxyresveratrol Drives Caspase-Independent Apoptosis-like Cell Death in MDA-MB-231 Breast Cancer Cells through the Induction of ROS. *Biochem. Pharmacol.* **2020**, *173*, 113724.
9. Dresch, R. R.; Haeser, A. S.; Lerner, C.; Mothes, B.; Vozári-Hampe, M. M.; Henriques, A. T. Detecção de Atividade Lectínica e Atividade Hemolítica Em Extratos de Esponjas (Porifera) Nativas Da Costa Atlântica Do Brasil. *Rev. Bras. Farmacogn.* **2005**, *15*, 16–22.

the Research and Drug Development Center, Fortaleza, Ceara, Brazil (CEUA – NPDM), under protocol number 41111022-0.

1, **4** and β-lapachone (1.56 - 200 μM) were tested for hemolytic activity according to Dresch et al. (2005)[9] in 96-well plates for 1 h at room temperature (25° C) using suspension of human erythrocytes (2% in 0.85% NaCl containing 10 mM $CaCl_2$). Triton X-100 1% was used as positive control. After centrifugation, hemoglobin levels in the supernatants were determined at 540 nm.

Assessment of the cell death mechanism

All experiments outlined below were performed using DU-145 cells to investigate the mechanism behind cytotoxicity. Furthermore, DU-145, a cell line characterized by overexpression of NQO1, was of particular interest in this research. For this purpose, the compounds were added to 24-well plates with cancer cells (0.4-0.7 × 104 cells/well) to obtain final concentrations of 2 and 4 μM for **1** and 0.5 and 1 μM for **4**. These concentrations were selected based upon the IC_{50} value for DU-145 cells determined by MTT assays after 24-hours exposure and correspond to the $IC_{50}/2$ and IC_{50}, as two different concentrations enabled to study concentration-dependent effect. β-lapachone (3 μM) was used as a positive control and DMSO 5% (vehicle) as a negative control. Three independent experiments performed in triplicate were conducted for all tests.

Trypan blue exclusion

Aliquots from each well were removed from cultures after 24 h treatment and cells scored in a Neubauer chamber using light microscopy (Metrimpex Hungary/PZO-Labimex Modelo Studar lab™).

Morphological examination of cell by light microscopy

Cells were seeded on sterile glass coverslips and treated for 24 hours, with DMSO as a negative control and β-lapachone (3 μM) as a positive control. After incubation, cells were fixed with 4% paraformaldehyde and stained with Giemsa solution (Laborclin Ltd, Paraná, Brazil). Observations were conducted using light microscopy (Olympus, Tokyo, Japan) at 200× magnification.

Flow cytometry analysis

Flow cytometry analysis was performed using a Guava easyCyte 6-2L flow cytometer (Merck Millipore), after 24 h of incubation with the naphthoquinones. Ten thousand events were evaluated per experiment.

Cell viability and membrane integrity: Cells were incubated with propidium iodide solution (50 μg/mL, Sigma™, USA) in the absence of light for 5 minutes. Fluorescence was measured and data were obtained on the number of cells and cell membrane integrity.

DNA Fragmentation: Cells were incubated with a lysis solution (Triton 100x 0,1% + RNAse 0,025%), therefore, the DNA content was analyzed with propidium iodide (50 μg/mL) in phosphate-buffered saline (PBS), in the absence of light for 30 minutes. After incubation, the fluorescence was measured and cell cycle was analyzed using the program ModFit LT version 6.0 (Verity Software House, Inc., Topsham, ME). The percentage of DNA fragmentation was determined according to the number of cells displaying subdiploid (sub-Go/G1) DNA in relation to the total number of cells examined.

Mitochondrial transmembrane potential (ΔΨm): Cells were incubated with rhodamine 123 (Rho-123) solution (1 μg/mL) in the absence of light at 37 °C for 15 minutes. Supernatant was withdrawn carefully, and the cells were resuspended in 300 μL of PBS and incubated at room temperature in the dark for another 30 minutes. Then, the samples were analyzed and the percentage of mitochondrial depolarization was determined.

Evaluation of Intracellular ROS

Reactive oxygen species (ROS) production was assessed using diacetate 5-(6)-chloromethyl-2',7'-dichlorodihydrofluorescein (CM-H2DCFDA) (KIM; XUE, 2020). Cells were seeded in 24-well plates (0.5 x 105 cells/well) and treated with **1** (4 μM) and **4** (1 μM) for 3, 6, and 24 hours to evaluate temporal effects. Following treatment, cells were incubated with CM-H2DCFDA (10 μM) for 30 minutes, then washed with PBS to remove excess probe. A Zeiss Axxio Observer microscope was used to image the cells at 200x magnification with 485 nm excitation and 535 nm emission. A minimum of three randomly selected fields per well were captured, and the presence of intracellular ROS was quantified using ImageJ software (National Institutes of Health, Bethesda, MD, USA). Hydrogen peroxide (200 μM) served as a positive control.

Evaluation of lipid peroxidation

The BODIPYTM 581/591 C11 undecanoic acid is a fluorescent fatty acid probe sensitive to lipid peroxidation assays, resulting in a shift in fluorescence emission peak from red (~590 nm), reduced state, to green (~510 nm) upon oxidation when exposed to oxidizing agents such as ROS.[10] Cells were seeded in 24-well plates on sterile glass coverslips (0.5 x 105 cells/well). The cells were treated with **1** (4 μM) and **4**

[10]. (a) Drummen, G. P. C.; van Liebergen, L. C. M.; Op den Kamp, J. A. F.; Post, J. A. C11-BODIPY581/591, an Oxidation-Sensitive Fluorescent Lipid Peroxidation Probe: (Micro)Spectroscopic Characterization and Validation of Methodology. *Free Radic. Biol. Med.* **2002**, *33*, 473–490. (b) Drummen, G. P. C.; Gadella, B. M.; Post, J. A.; Brouwers, J. F. Mass Spectrometric Characterization of the Oxidation of the Fluorescent Lipid Peroxidation Reporter Molecule C11-BODIPY581/591. *Free Radic. Biol. Med.* **2004**, *36*, 1635–1644.

(1 μM) for 6 hours, followed by a 30-minute incubation with BODIPY™ (10 μM). After washing with PBS, cells were fixed with 10% paraformaldehyde and stained with Hoechst (1:2000 in PBS) for 10 minutes in the dark. Following two additional washes with PBS, coverslips were mounted to preserve fluorescence. Fluorescence microscopy was performed using a Zeiss Axxio Observer microscope at 200x magnification, capturing a minimum of three randomly selected fields per well for analysis using ImageJ software (National Institutes of Health, Bethesda, MD, USA).

Analysis of DNA strand breaks (Comet assay)

The comet assay was conducted using two variations: alkaline and neutral, following the modified protocols of Calvacanti (2020) and recommendations from the International Workshop on Genotoxicity Test Procedures.[11] The involvement of glutathione-dependent enzymes was assessed by including N-Acetyl-Cysteine (NAC) in the treatments. Following treatment, cells were washed, trypsinized, and mixed with 0.75% low melting point agarose on slides pre-coated with 1% regular melting point agarose. After solidification, slides were immersed in a lysis solution (comprising 2.5 M NaCl, 10 mM Tris, 100 mM EDTA, 1% Triton X-100, and 10% DMSO at pH 10) for 1 h to extract DNA. For the alkaline assay, electrophoresis was conducted in an alkaline buffer 300 mM NaOH and **1** mM EDTA at pH > 13) for 20 minutes at 25 V and 300 mA (0.86 V/cm) to reveal alkali-labile sites, while the neutral assay utilized a different buffer and conditions. The neutral protocol followed a similar procedure with adjustments to the pH conditions. Electrophoresis for the neutral version was carried out in a buffer comprising 100 mM Tris and 300 mM sodium acetate at pH 8.5 for 60 minutes at 12 mA and 14 V (0.5 V/cm), preceded by a 60-minute equilibrium period in the same buffer.

In both versions of the comet assay, the slides underwent neutralization using 0.4 M Tris at pH 7.5. For each concentration, 100 randomly selected cells were scored into five damage classes, yielding a damage index from 0 (undamaged) to 400 (maximally damaged).[11] Additionally, co-incubation with NAC and human 8-hydroxyguanine DNA-glycosylase (hOGG1) was performed to explore the relationship between oxidative stress and DNA damage. NAC replenishes reduced glutathione levels, protecting cells from oxidative stress, while hOGG1 enhances the comet assay's sensitivity to oxidative DNA damage, particularly 8-oxoguanine and methyl-fapy-guanine.[12]

---

11. (a) Cavalcanti, B. C.; Andrade Neto, J. B.; de Sousa Silva, A. A.; Barreto, F. S.; de Oliveira Ferreira, J. R.; Rocha da Silva, C.; Aires do Nascimento, F. B. S.; do Amaral Valente Sá, L. G.; Ferreira Magalhães, H. I.; Nobre Júnior, H. V.; de Moraes, M. O. Evaluation of Genotoxicity and Mutagenicity of Ketamine on Human Peripheral Blood Leukocytes and in Salmonella Typhimurium. *Toxicol. In Vitro.* **2020**, *62*, 104718. (b) Tice, R. R.; Agurell, E.; Anderson, D.; Burlinson, B.; Hartmann, A.; Kobayashi, H.; Miyamae, Y.; Rojas, E.; Ryu, J. C.; Sasaki, Y. F. Single Cell Gel/Comet Assay: Guidelines for in Vitro and in Vivo Genetic Toxicology Testing. *Environ. Mol. Mutagen.* **2000**, *35*, 206–221.
12. Smith, C. C.; O'Donovan, M. R.; Martin, E. A. HOGG1 Recognizes Oxidative Damage Using the Comet Assay with Greater Specificity than FPG or ENDOIII. *Mutagenesis* **2006**, *21*, 185–190.

Investigation of the involvement of the NQO1 enzyme

MTT Assay

To explore the role of this enzyme in the mechanism of action or neutralization of the selected compounds, these compounds were tested on cell lines with high NQO1 expression (DU-145) and low or no expression (HL-60), in both the presence and absence of dicoumarol (50 μM), a known NQO1 inhibitor. Cell lines were chosen based on their expression profiles from the Human Protein Atlas database and the viability was assessed as described above using MTT assay.

Relative enzymatic activity

The reaction mixture contained 8 nM of NQO1 protein, 2000 nM of either **1**, **4**, or β-Lapachone, dissolved in 10 mM phosphate buffer (pH 7.5). The complete reaction medium included 10 mM phosphate buffer, 200 μM NADH, 0.07% w/v bovine serum albumin, 0.2% v/v Tween-20, and 0.2 mg/mL MTT, prepared immediately before the experiment and kept on ice, protected from light. In each well, 50 μL of PBS, protein, tested compounds, and reaction medium were added sequentially to initiate the reaction. After 5 minutes, absorbance was measured at 595 nm using a VICTOR™ NOVO plate reader (PerkinElmer).

Thermal Shift Assay

The Thermal Shift assay was conducted to monitor the thermal denaturation of the NQO1 protein by increasing the temperature while continuously measuring the fluorescence emission of SYPRO Orange. This assay quantifies the stabilization of the protein in the presence of tested ligands, indicated by an increase in the melting temperature.[13] A 96-well PCR plate was used, and the reaction mixtures included 50 mM phosphate buffer (pH 7.5 with 150 mM NaCl), 100 μM of ligands (**1**, **4**, or β-lapachone), 10X SYPRO Orange fluorophore, and 10 μM recombinant NQO1 protein. Dicoumarol (100 μM) served as a positive control. Each solution was prepared to a total volume of 200 μL, and the denaturation temperature was monitored from 25 to 95 °C using a C1000 Touch thermocycler and CFX96 Real-Time system (BIO-RAD).

---

[13]. Matulis, D.; Kranz, J. K.; Salemme, F. R.; Todd, M. J. Thermodynamic Stability of Carbonic Anhydrase: Measurements of Binding Affinity and Stoichiometry Using ThermoFluor. *Biochemistry.* **2005**, *44*, 5258–5266.

Molecular Docking

To perform molecular modelling, the structure of the human NAD(P)H Quinone Oxidoreductase **1** (NQO1) (PDB 2F1O), was retrieved from the Protein Data Bank protein database. Molecular docking analyzes were performed using the AutoDock Vina code (version 1.1.2), using the Lamarckian Genetic Algorithm and 3 multithreading paths.[14] The grid box was centralized throughout the enzyme considering the parameters 122 Å × 114 Å x 126 Å and dimensions (x, y, z) = (1.758, -10.118, 18.969). Simulations were independently carried out with 50 poses each as a standard procedure. The codes UCSF Chimera[15] and Discovery Studio Visualizer (Dassault Systemes Biovia, 2019) were used to examine the outcomes.

Quantum biochemistry simulations

Interaction energy calculations were conducted using quantum biochemistry methods for Dicumarol (DIC, $C_{19}H_{12}O_6$, PubChem CID: 54676038) and the compound **4**. For DIC, the crystallographic structure of the human enzyme NQO1 cocrystallized with the ligand (PDB ID: 2F1O)[16] served as the starting point. Hydrogens were added to this structure, followed by constrained optimization of their positions, while keeping the heavy atoms of both the protein and the ligand fixed.

For **4**, the best-fitting conformation obtained from previously described molecular docking studies was utilized, demonstrating binding at the same site as DIC in NQO1. Only **4** and the hydrogens of NQO1 were allowed to move freely.

Data preparation was carried out using the Discovery Studio Visualizer software, which facilitated the addition of hydrogens and the definition of formal charges. Geometric optimizations were performed in Forcite software, employing the UNIVERSAL force field with ultra-fine convergence criteria and a cutoff radius of 18.5 Å, utilizing cubic spline truncation.

The interaction energies between the ligands and each amino acid residue of both the A and C chains of the NQO1 protein were estimated using an adaptation of the MFCC (Molecular Fragmentation with Conjugate Caps) method.[17] To determine the interaction energy between NQO1 and the ligands, we

---

14. Trott, O.; Olson, A. J. AutoDock Vina: Improving the Speed and Accuracy of Docking with a New Scoring Function, Efficient Optimization, and Multithreading. *J. Comput. Chem.* **2010**, *31*, 455–461

15. Pettersen, E. F.; Goddard, T. D.; Huang, C. C.; Couch, G. S.; Greenblatt, D. M.; Meng, E. C.; Ferrin, T. E. UCSF Chimera—A Visualization System for Exploratory Research and Analysis. *J. Comput. Chem.* 2004, *25*, 1605–1612.

16. Asher, G.; Dym, O.; Tsvetkov, P.; Adler, J.; Shaul, Y. The Crystal Structure of NAD(P)H Quinone Oxidoreductase 1 in Complex with Its Potent Inhibitor Dicoumarol. *Biochemistry.* **2006**, *45*, 6372–6378.

17. da Costa, R. F. Losartan as an ACE Inhibitor: A Description of the Mechanism of Action through Quantum Biochemistry. *RSC Adv.* **2022**, *12*, 28395–28404. (b) da Costa, R. F.; Freire, V. N.; Bezerra, E. M.; Cavada, B. S.; Caetano, E. W. S.; de Lima Filho, J. L.; Albuquerque, E. L. Explaining Statin Inhibition Effectiveness of HMG-CoA Reductase by Quantum Biochemistry Computations. *Phys. Chem. Chem. Phys.* **2012**, *14*, 1389–1398. (c) Dantas, D. S.; Oliveira, J. I. N.; Lima Neto, J. X.; da Costa, R. F.; Bezerra, E. M.; Freire, V. N.; Caetano, E. W. S.; Fulco, U. L.; Albuquerque, E. L. Quantum Molecular Modelling of Ibuprofen Bound to Human Serum Albumin. *RSC Adv.* **2015**, *5*, 49439–49450. (d) Farias, K.; da Costa, R. F.; Meira, A. S.; Diniz-Filho, J.; Bezerra, E. M.; Freire, V. N.; Guest, P.; Nikahd, M.; Ma, X.; Gardiner, M. G.; Banwell, M. G.; de Oliveira, M. C. F.; de Moraes, M. O.; Pessoa, C. do Ó. Antitumor Potential of the Isoflavonoids (+)- and (−)-2,3,9-Trimethoxypterocarpan: Mechanism-of-Action Studies. *ACS Med. Chem. Lett.* **2020**, *11*, 1274–1280.

designated the caps as the amino acid residues preceding and following the protein's peptide chain sequence. The interaction energy between the ligand and each individual amino acid residue of the protein was calculated according to the equation provided below:[16]

$$\boldsymbol{E(L - R_i)} = \underbrace{\boldsymbol{E(L + R^*_{i-1}R_iR_{i+1})}}_{E_A} - \underbrace{\boldsymbol{E(R^*_{i-1}R_iR_{i+1})}}_{E_B} - \underbrace{\boldsymbol{E(L + R^*_{i-1} + R_{i+1})}}_{E_C} + \underbrace{\boldsymbol{E(R^*_{i-1} + R_{i+1})}}_{E_D}$$

To facilitate the generation of input files, a Perl script was developed. Energy calculations were conducted using the $DMol^3$ program, which is based on Density Functional Theory (DFT) and employs the local density approximation (LDA). The Perdew-Wang functional (PWC) was utilized, incorporating OBS dispersion correction and the Double Numerical plus Polarization (DNP) basis set. A dielectric constant of 40 was adopted to simulate the biological environment, which is a common practice in computational biology to account for the screening effect of the solvent on the electrostatic interactions.[17]

Statistical analysis

Each experiment was conducted independently and values are expressed as the mean value ± standard error of the mean (SEM). The IC_{50} values were obtained from the nonlinear regression of the cytotoxicity curves. Statistical analyses for multiple comparisons were performed using one-way ANOVA followed by Tukey’s post-hoc test. $p < 0.05$ was considered statistically significant.

Quantum biochemistry simulations

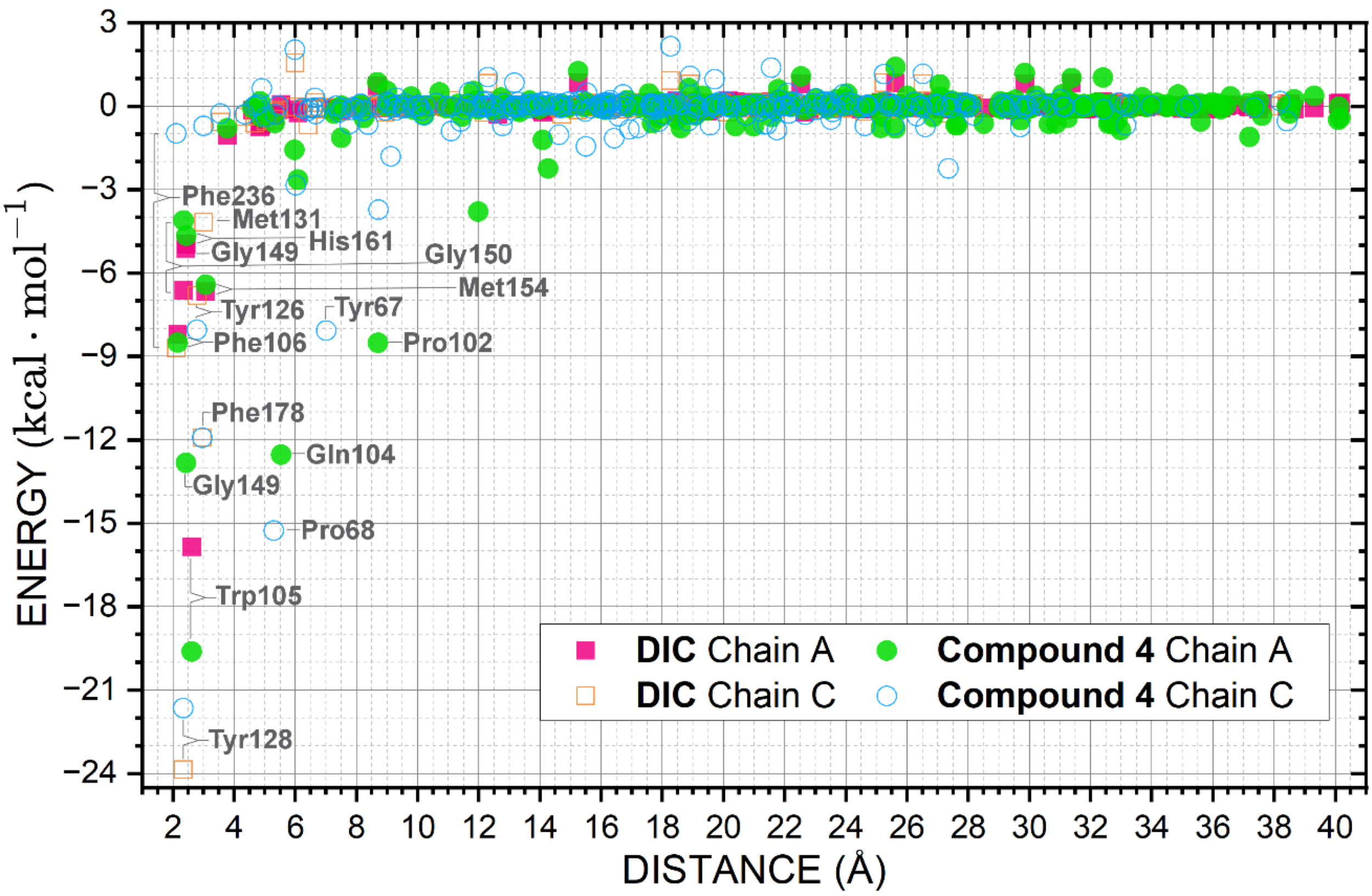

Figure S1. Distribution of interaction energies as a function of the distance between the amino acids of NQO1 enzyme and the ligands dicoumarol (DIC, squares) and **4** (circles). The vertical axis represents the interaction energy (kcal mol^{-1}), and the horizontal axis shows the distance (Å) between the amino acids and the ligands. Interactions with chain A are indicated by pink squares (DIC) and green circles (**4**), both of which are filled. Interactions with chain C are indicated by orange squares (DIC) and blue circles (**4**), both of which are unfilled. The residues that contribute most to ligands stabilization are highlighted in the plot. The theoretical results were obtained using DFT/LDA/PWC, OBS dispersion correction, and the DNP basis set. To simulate the biological environment, a dielectric constant of 40 was adopted.

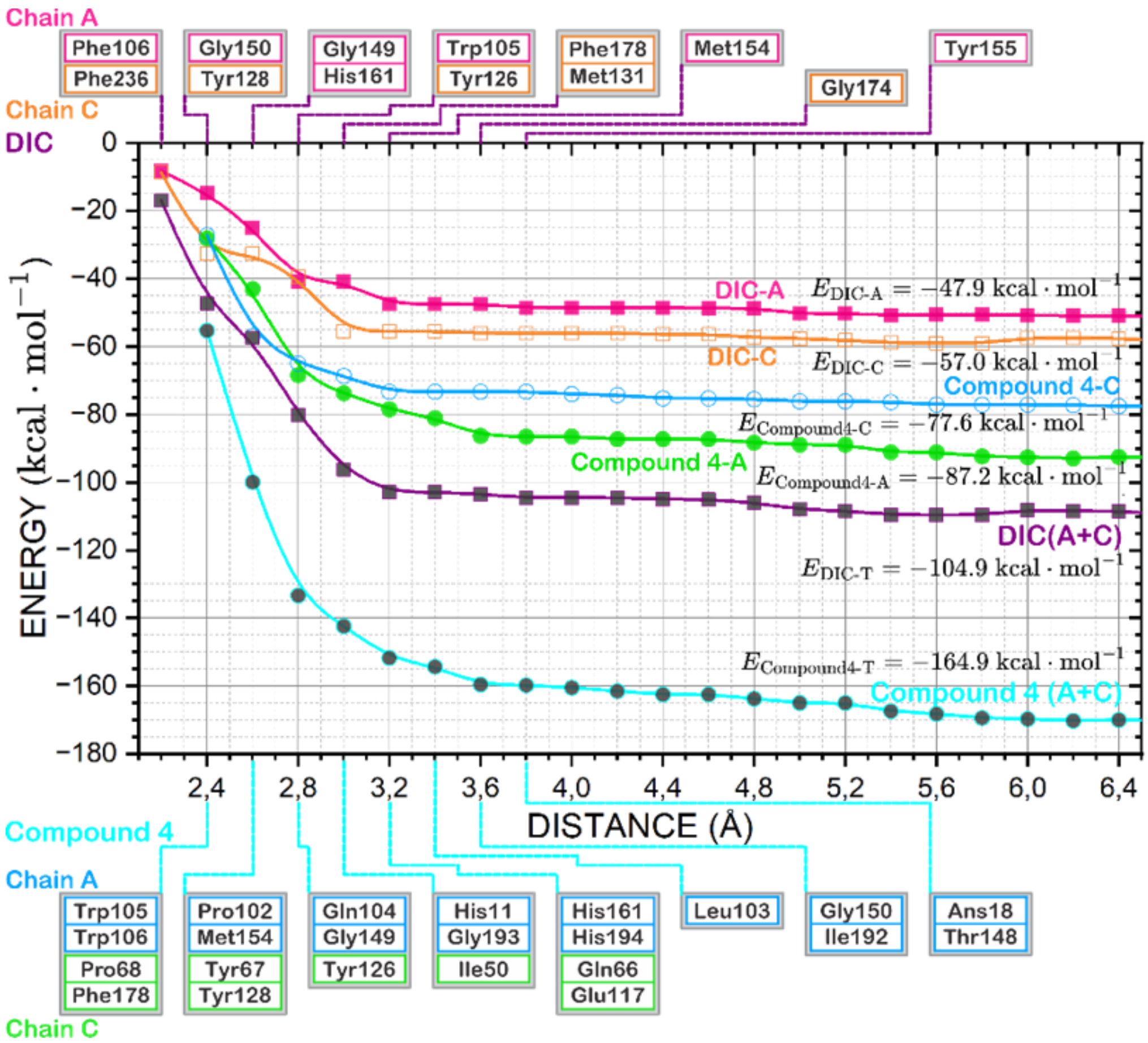

Figure S2. Interaction energy profile between the ligands Dicoumarol (DIC) and **4** and the amino acids of chains A and C of NQO1 enzyme as a function of distance. The curves represent the cumulative sum of the interaction energies of residues located within 0.2 Å intervals. Interactions with chain A are indicated by pink squares (DIC-A) and green circles (**4-A**), while interactions with chain C are represented by orange squares (DIC-C) and blue circles (**4-C**), all connected by lines. The total energy curves for each ligand are highlighted with filled gray symbols: purple squares for DIC and cyan circles for **4**.

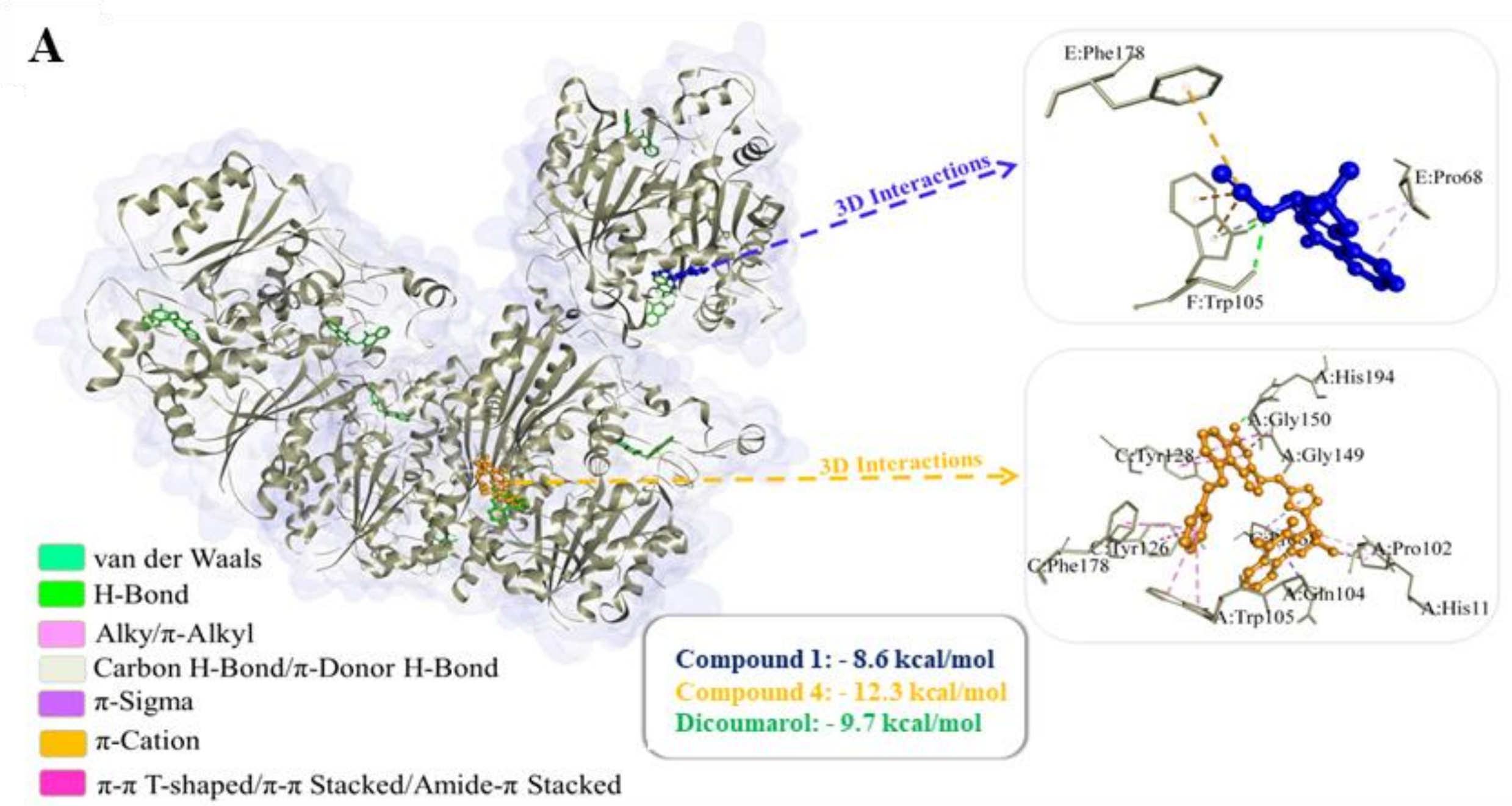

B

Protein	PDBID	Compound	Affinity (kcal/mol)
		Compound **1**	-8.6
**NQO1**	**2F1O**	Compound **4**	-12.3
		Dicoumarol	7.97

Figure S3. Structures of the probable complexes formed between **1**, **4** and dicoumarol with the target NQO1 (PDB ID: 2F1O).

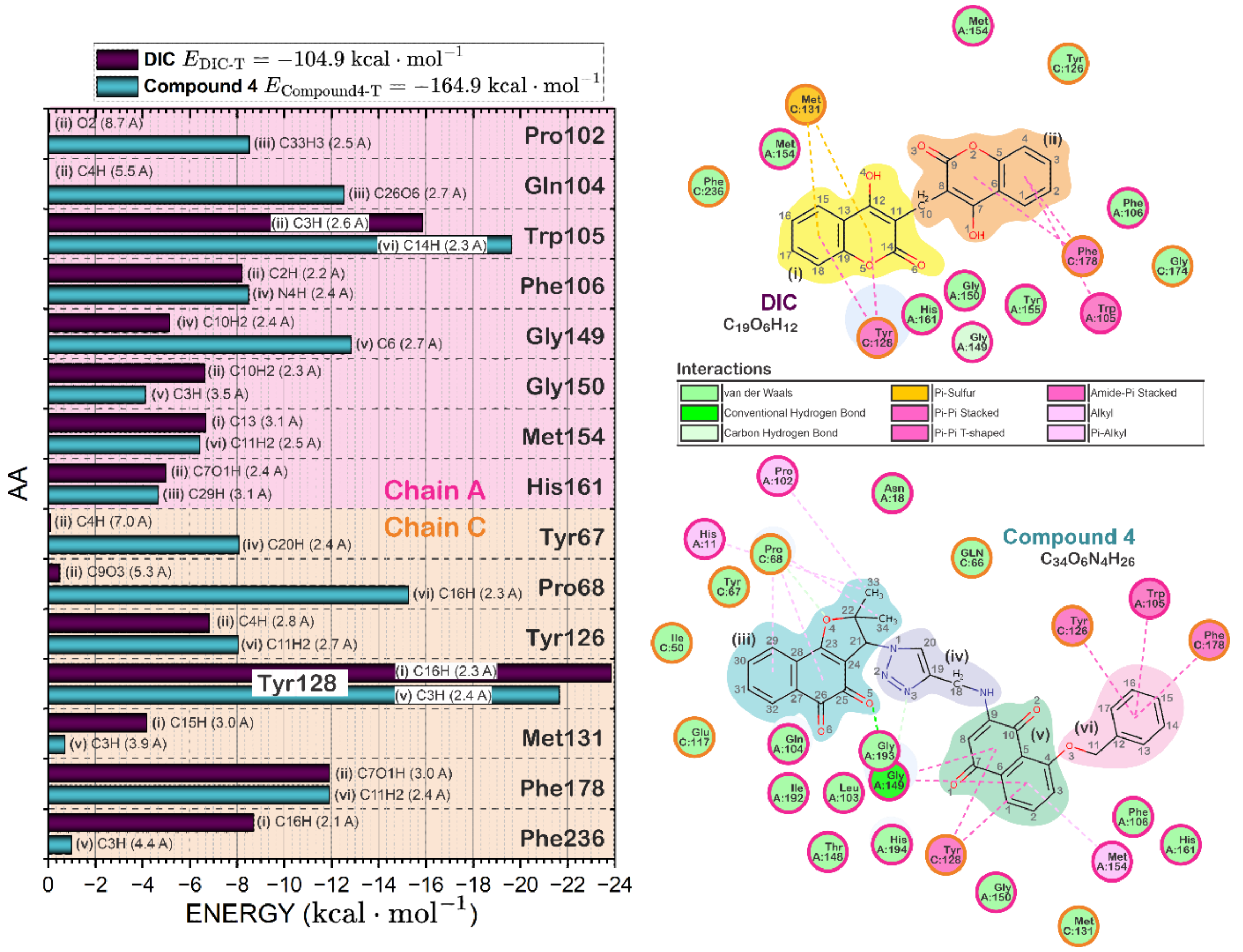

Figure S4. BIRD panel (Binding Site, Interaction Energy, and Residue Domain) shows the key residues from chains A (pink area) and C (orange area) of the NQO1 protein, highlighting their interaction energy contributions with the ligands DIC (purple bars) and **4** (light blue bars). The closest atoms of DIC and **4** to each residue are indicated next to the bars, along with their respective distances (in Å). On the right, the 2D interaction diagram displays the residues from chains A (pink border) and C (orange border), along with their interactions with the ligands, specifying the types of molecular interactions observed. The planar structures of DIC and **4** are subdivided into interaction fragments: "i" (yellow) and "ii" (orange) for DIC; "iii" (blue), "iv" (purple), "v" (green), and "vi" (pink) for **4**. Atoms are labeled to facilitate analysis of their contacts with the NQO1 target protein. The figure was generated using Discovery Studio Visualizer (Discovery Studio Modeling Environment, Version 19.1; https://www.accelrys.com).

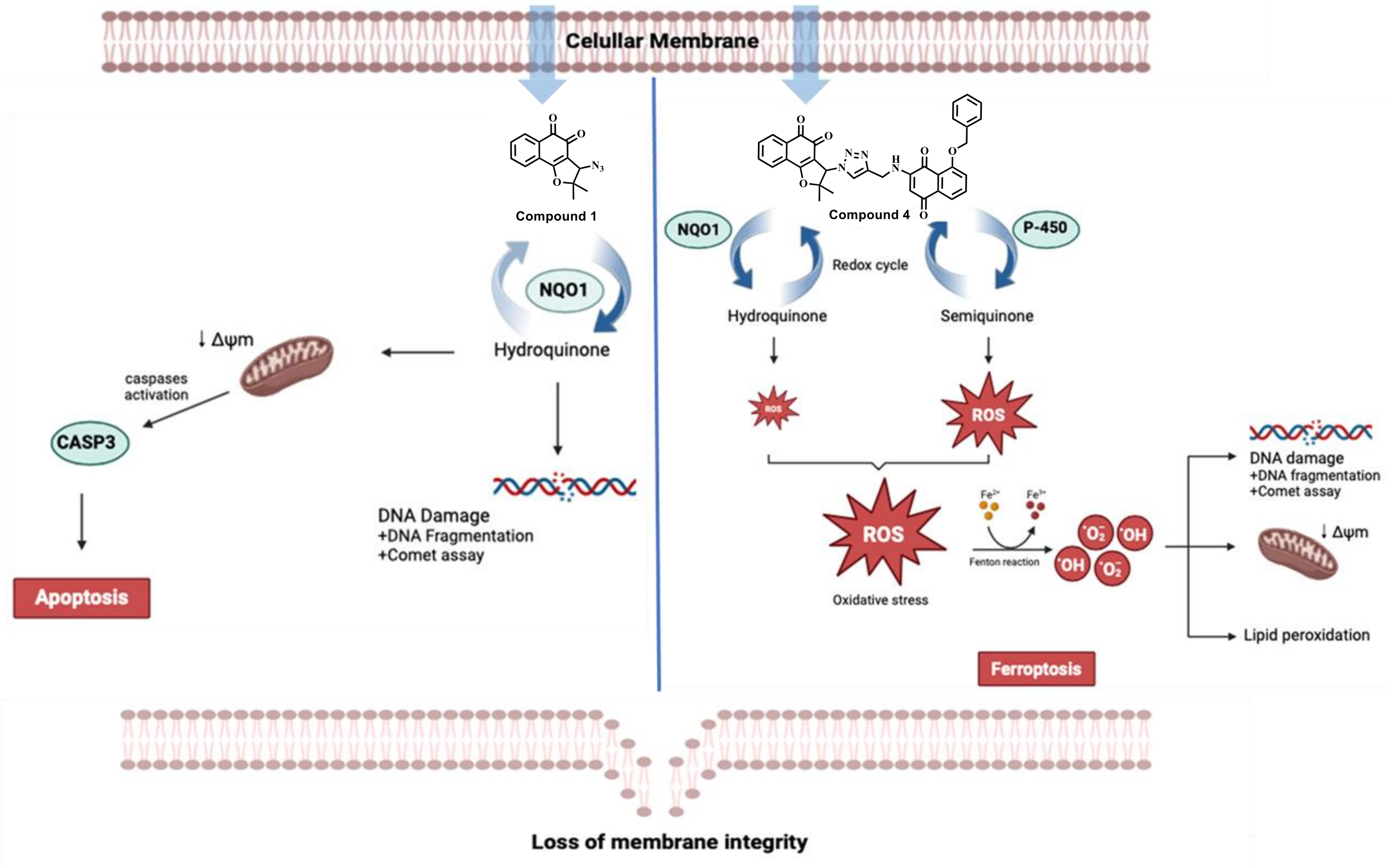

Figure S5. General view of the mechanism of action of **1** and **4** in DU-145 cells. Created using Biorender.com.

^{1}H and ^{13}C NMR Spectra

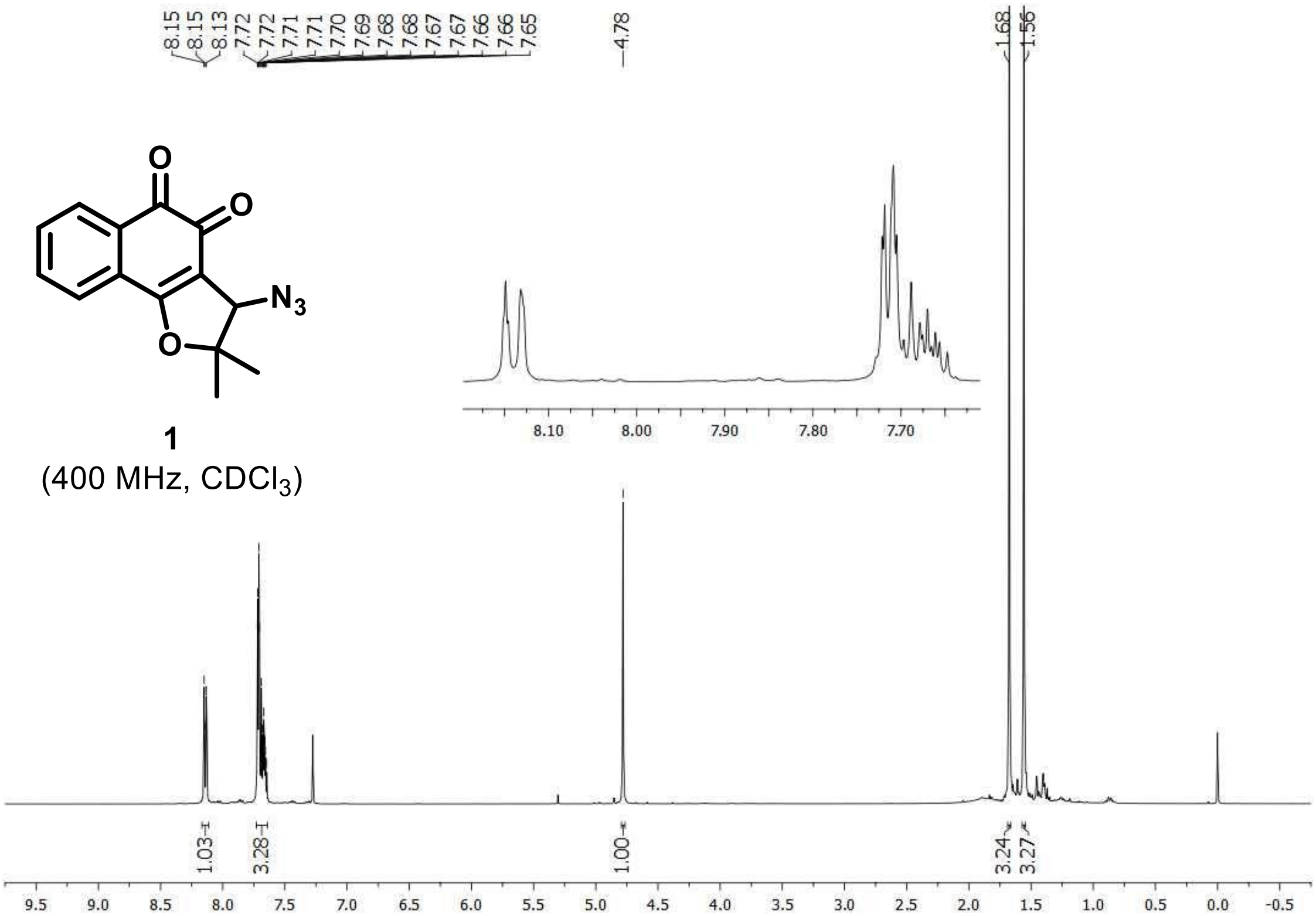

Figure S6. ^{1}H NMR spectrum of compound **1.**

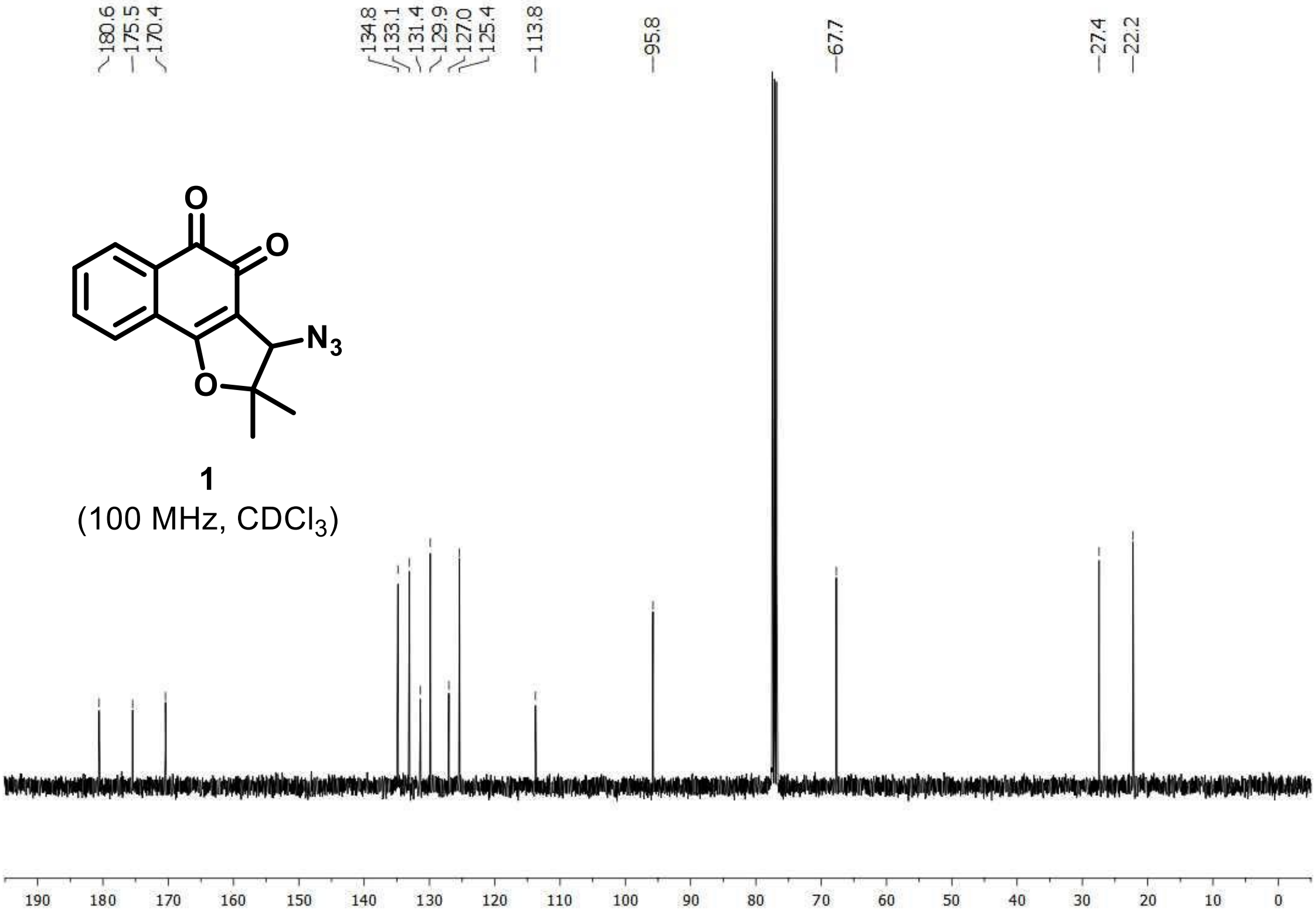

Figure S7. ^{13}C NMR spectrum of compound **1.**

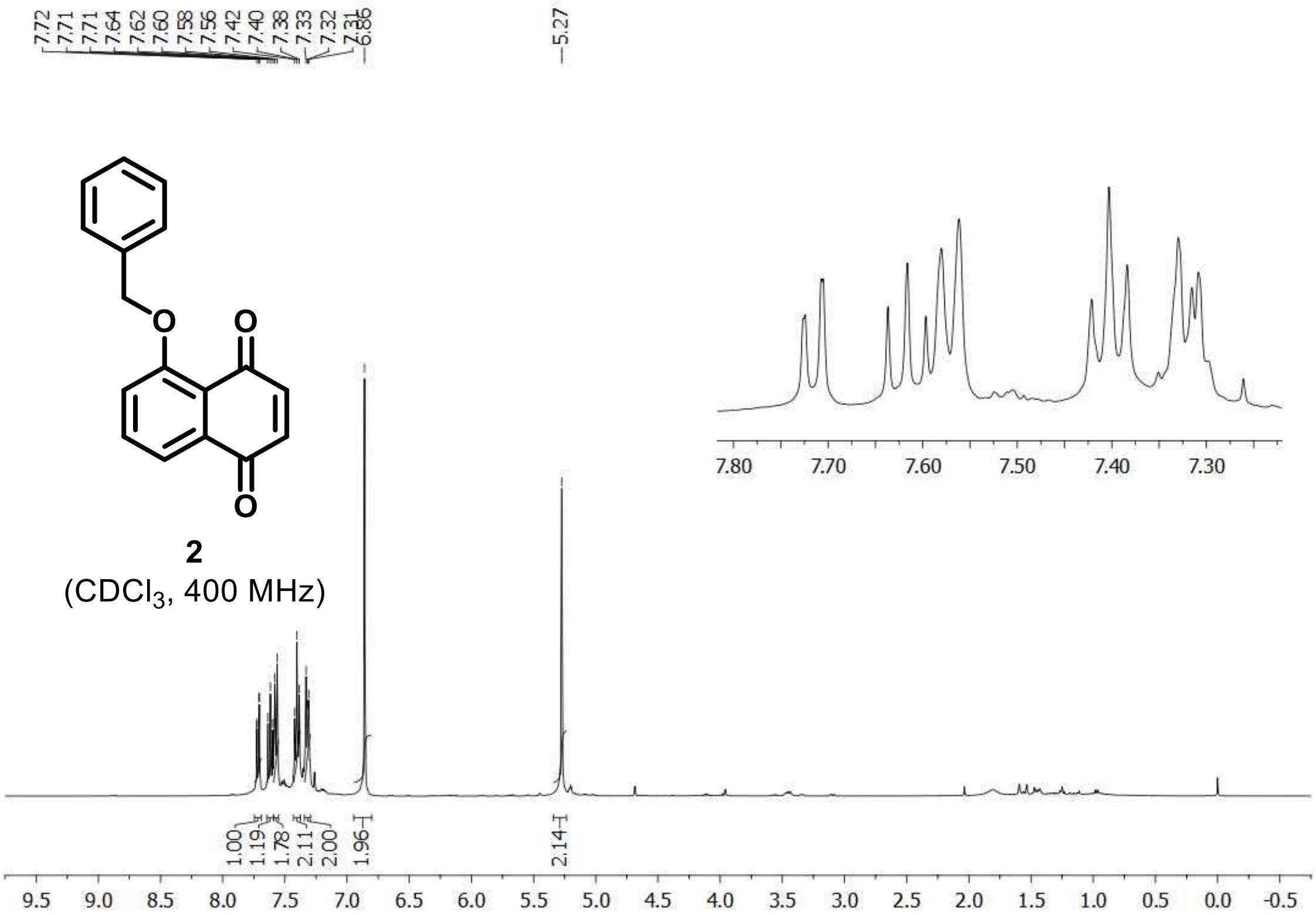

Figure S8. ^{1}H NMR spectrum of compound **2.**

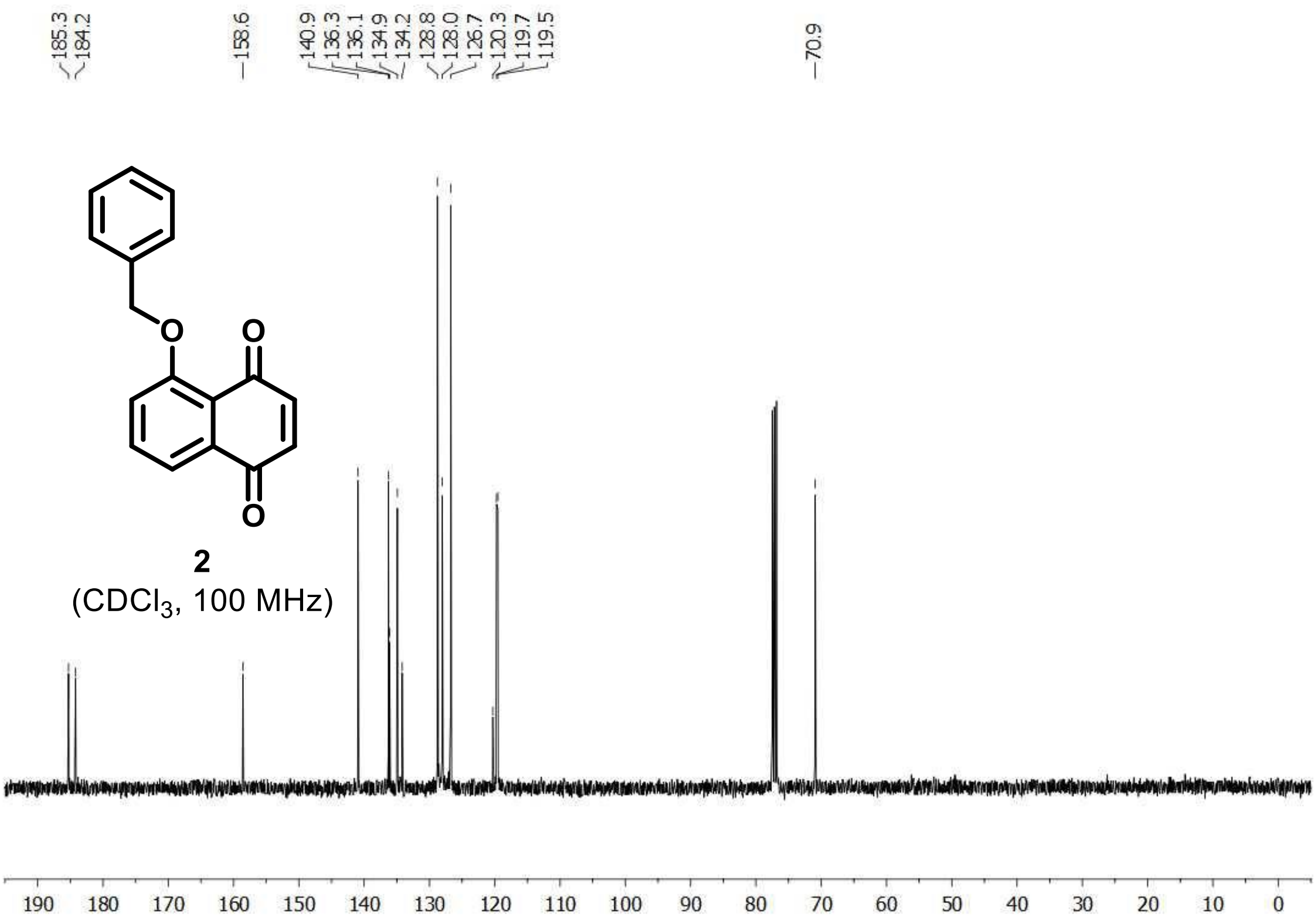

Figure S9. ^{13}C NMR spectrum of compound **2.**

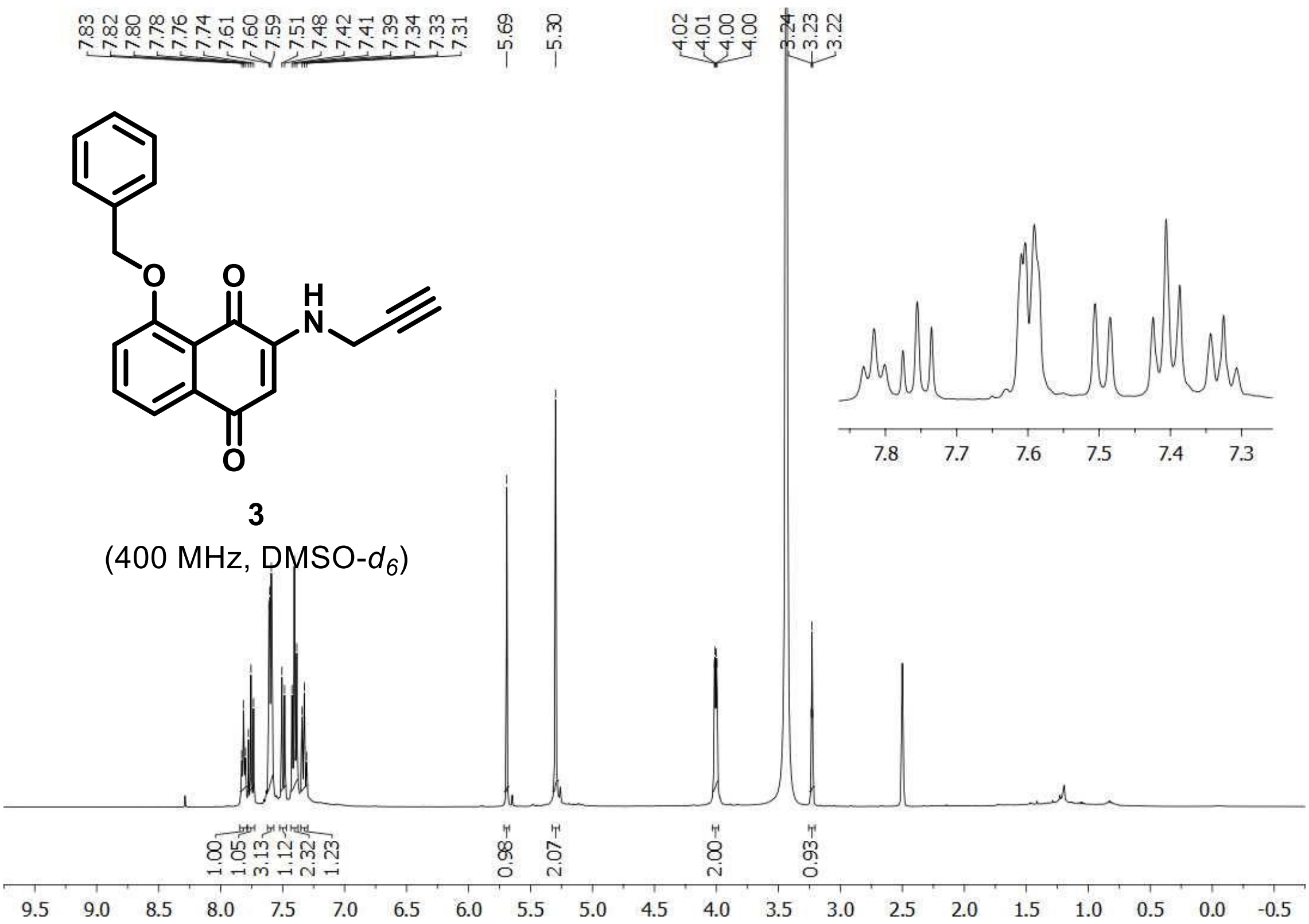

Figure S10. ^{1}H NMR spectrum of compound **3.**

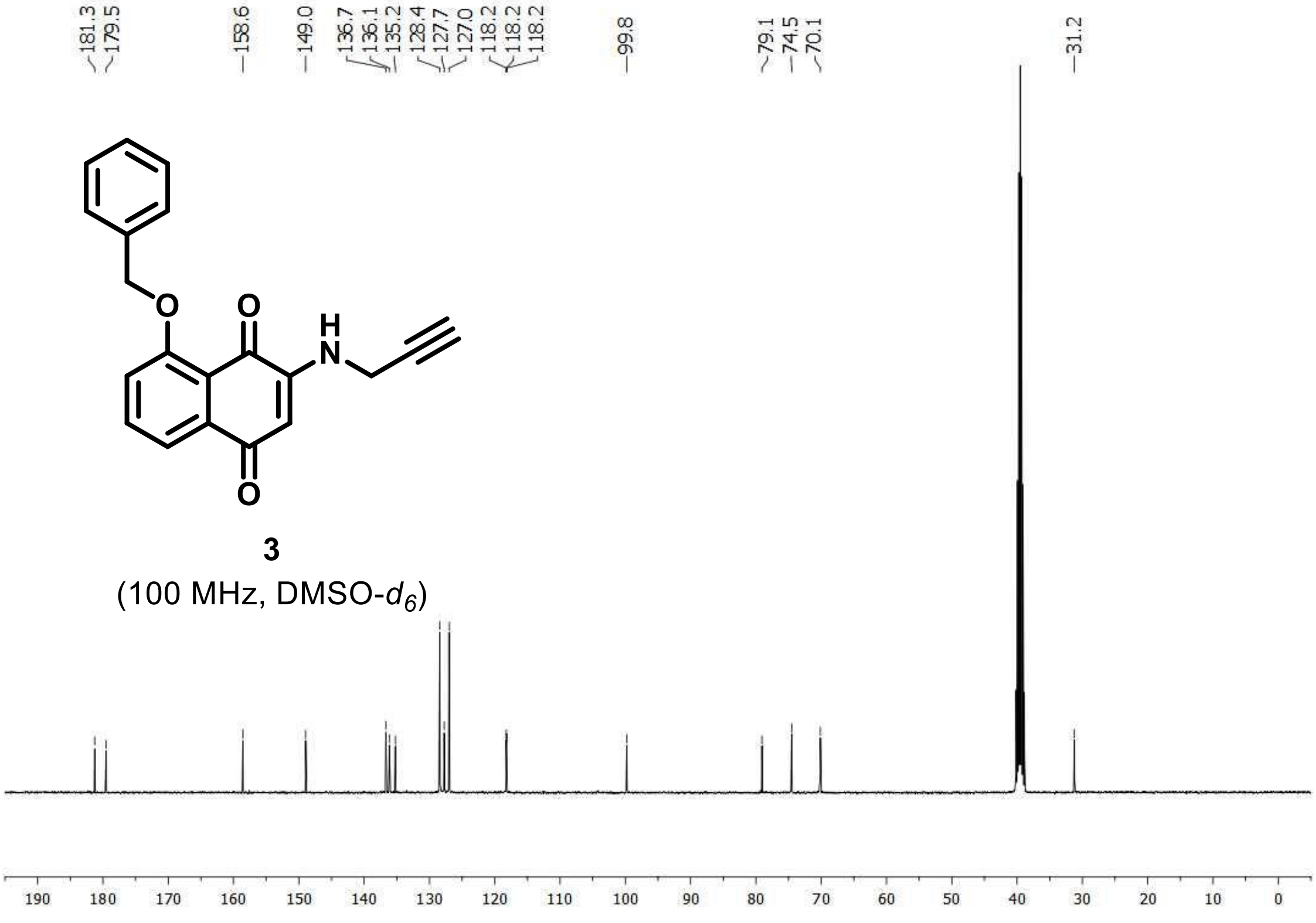

Figure S11. ^{13}C NMR spectrum of compound **3.**

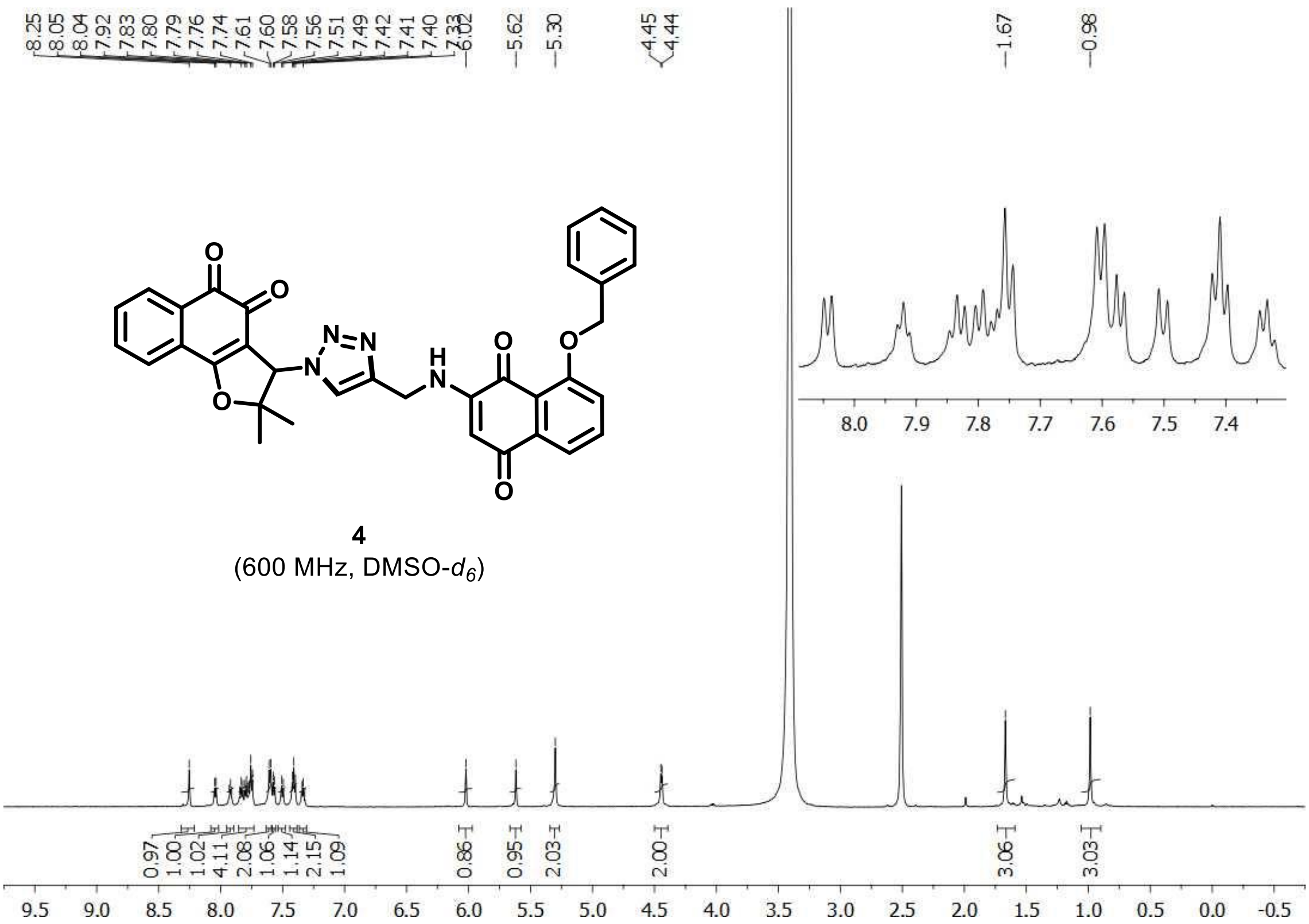

Figure S12. ^{1}H NMR spectrum of compound **4.**

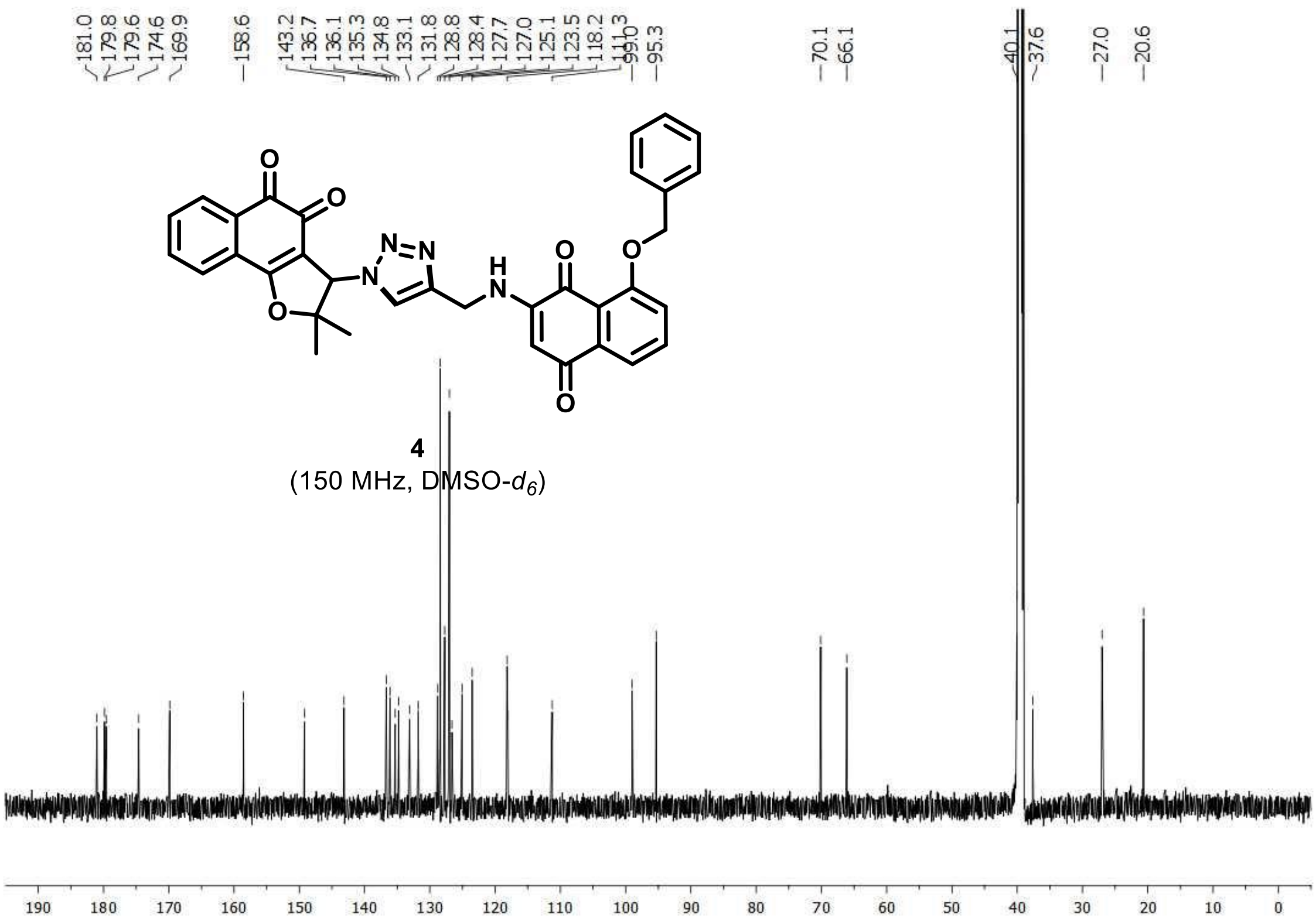

Figure S13. ^{13}C NMR spectrum of compound **4.**

ESI+ | Sample dissolved in DMSO and diluted in MeOH:H_2O (1:1) with 0.1% formic acid.

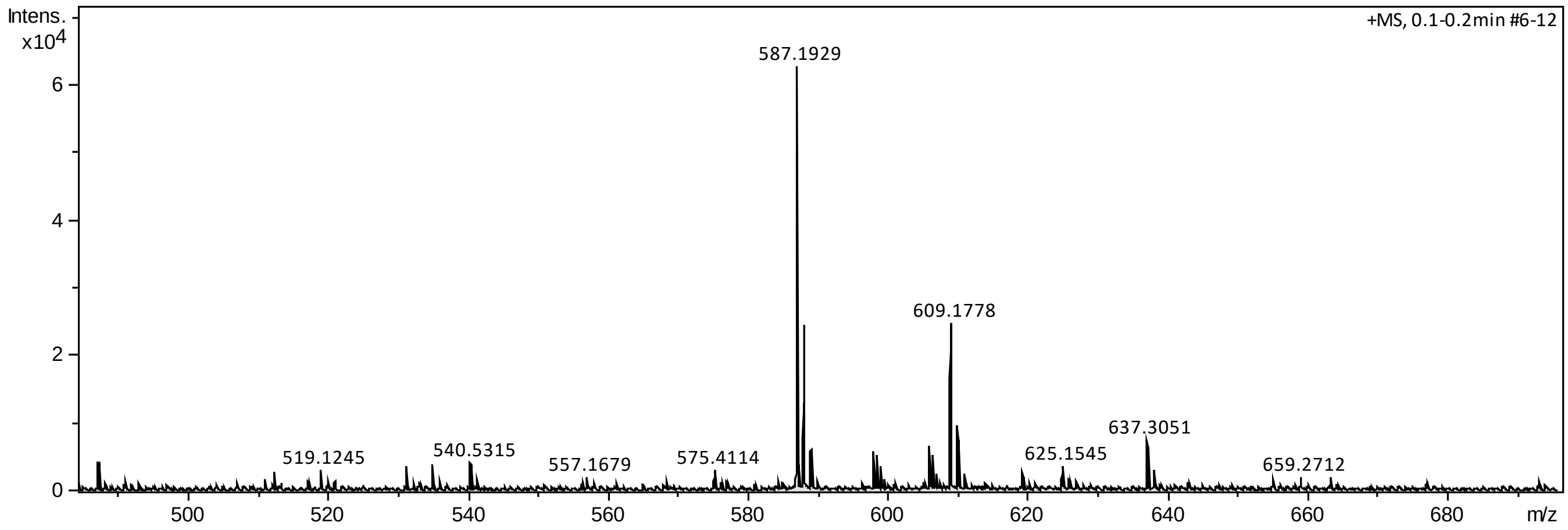

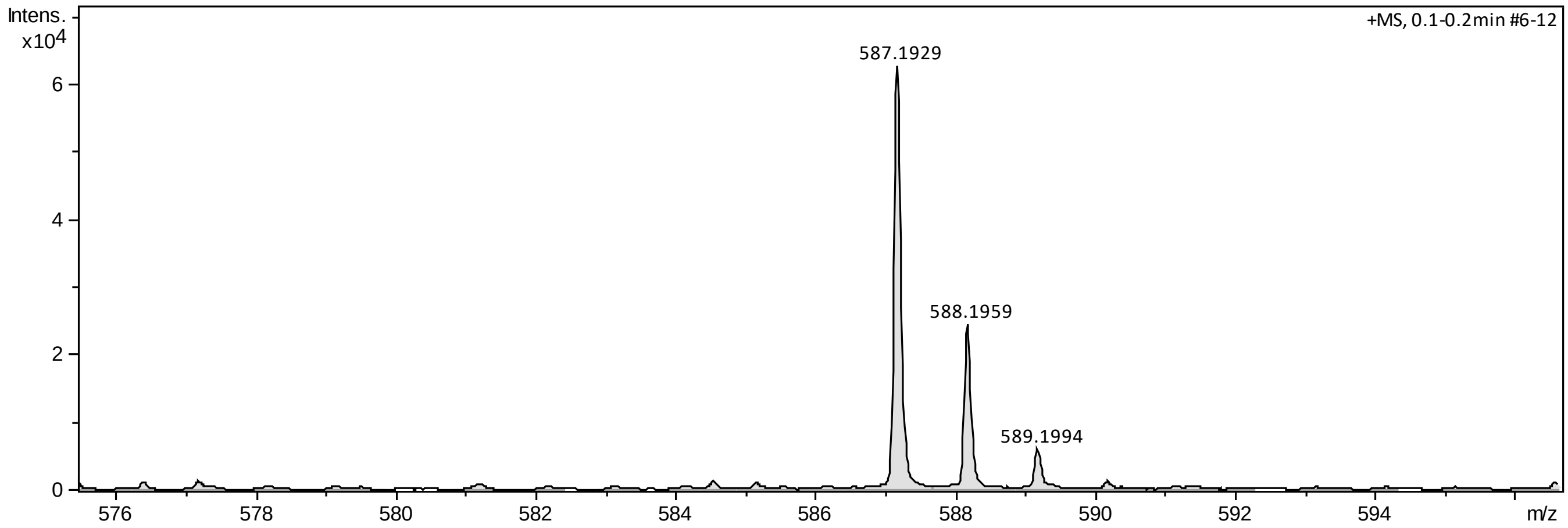

Figure S14. HRMS spectrum of compound **4.**
